# Modulation of Extrinsic and Intrinsic Signaling Together with Neuronal Activation Enhances Forelimb Motor Recovery after Cervical Spinal Cord Injury

**DOI:** 10.1523/ENEURO.0359-24.2025

**Published:** 2025-02-28

**Authors:** Hirohide Takatani, Naoki Fujita, Fumiyasu Imai, Yutaka Yoshida

**Affiliations:** ^1^Neural Connectivity Development in Physiology and Disease Laboratory, Burke Neurological Institute, White Plains, New York 10605; ^2^Laboratory of Veterinary Surgery, Graduate School of Agriculture and Life Sciences, The University of Tokyo, Bunkyo-ku, Tokyo 113-0032, Japan; ^3^Brain and Mind Research Institute, Weill Cornell Medicine, New York 10065; ^4^Neural Circuit Unit, Okinawa Institute of Science and Technology Graduate University, Onna-son, Kunigami-gun, Okinawa 904-0495, Japan

**Keywords:** corticospinal tract, hM3Dq, Pten, RhoA, spinal cord injury

## Abstract

Singular strategies for promoting axon regeneration and motor recovery after spinal cord injury (SCI) have been attempted with limited success. For instance, the deletion of *RhoA* and phosphatase and tensin homolog (*Pten*) (an extrinsic and intrinsic modulating factor, respectively) in corticospinal neurons (CSNs) promotes axon sprouting after thoracic SCI; however, it is unable to restore motor function. Here, we examine the effects of combining *RhoA/Pten* deletion in CSNs with chemogenetic neuronal stimulation on axonal growth and motor recovery after SCI in mice. We find that this combinatorial approach promotes greater axonal growth and presynaptic bouton formation in CSNs within the spinal cord compared with *RhoA*;*Pten* deletion alone. Furthermore, chemogenetic neuronal stimulation of *RhoA*;*Pten*-deleted CSNs improves forelimb performance in behavioral tasks after SCI compared with *RhoA*;*Pten* deletion alone. These results demonstrate that combination therapies pairing genetic modifications with neuronal stimulation can promote greater presynaptic formation and motor recovery following SCI than either strategy alone.

## Significance Statement

In this study, we examined whether pairing neural stimulation with genetic deletion of *RhoA* and *Pten* in corticospinal neurons (CSNs) would enhance axonal growth and motor recovery after spinal cord injury (SCI). We found that this combinatorial approach, relative to singular strategies, promoted greater presynaptic bouton formation in injured CSNs in the spinal cord, resulting in improved motor recovery in those mice. These results open the possibility for even greater enhancements in healing and recovery following SCI through carefully crafted, multifaceted treatments tailored to address the specific needs of individual SCI patients.

## Introduction

Recovery of motor function after spinal cord injury (SCI) is likely to require substantial axonal growth and neural rewiring. In the adult central nervous system, full recovery from SCI is rarely observed. The corticospinal tract (CST), the major descending tract that controls voluntary movements, conveys motor commands from the sensorimotor cortex to the spinal cord, making it an important target for axon regeneration following SCI (Porter and Lemon, 1993; Oudega and Perez, 2012; Nicola et al., 2022). Although numerous potential therapeutic methods have been attempted, no single approach has fully reconstructed the injured CST or restored motor function after SCI. We propose that a combinatorial strategy targeting signaling pathways and neural activity may yield superior results compared with individual therapies.

Several molecules, such as phosphatase and tensin homolog (Pten), KLF7, Rho, and Sox11, have been shown to prevent axon regeneration after SCI (Liu et al., 2010; Blackmore et al., 2012; Fujita and Yamashita, 2014; Wang et al., 2015). Pten is an enzyme that suppresses the mechanistic target of rapamycin (a critical signaling component for axon growth), which prevents new protein synthesis and neuronal growth (Zhang et al., 2018). Deletion of *Pten* promotes significant regeneration of corticospinal (CS) axons after SCI, facilitating robust extension of axons across the lesion site (Liu et al., 2010). In addition, the Rho family of small GTPases, including RhoA, are important regulatory signaling molecules that are triggered by extrinsic molecules, such as myelin-associated inhibitors, repulsive axon guidance molecules, and inhibitory extracellular matrix molecules after SCI (Fujita and Yamashita, 2014). The Rho signaling pathway is involved in actin cytoskeletal dynamics that can lead to growth cone collapse and axon growth inhibition. Pharmacological Rho inhibitors enhance sprouting and regeneration of CS axons after SCI, but the extent of regeneration is variable (Fournier et al., 2003; Duffy et al., 2009; Boato et al., 2010; Fujita and Yamashita, 2014). It has already been shown that co-deletion of *RhoA* and *Pten* from the sensorimotor cortex prior to SCI resulted in reduced CS axonal dieback and enhanced rewiring of CS circuits to the spinal cord and hindlimb muscles; however, this approach to changing the internal growth state of CS neurons (CSNs) alone did not promote axon regrowth through the lesion and did not enhance hindlimb motor recovery after SCI (Nakamura et al., 2021). A method for eliciting and strengthening novel CS circuits may be required for capitalizing on the enhanced axonal plasticity resulting from *RhoA* and *Pten* deletion.

Neuronal stimulation is a robust approach for driving CS circuit plasticity that may promote motor recovery. Previous studies indicate that electrical stimulation of the cortex can transform neuronal circuits from a nonfunctional to a highly functional state and promote extensive sprouting of CSNs which restores neurological function after SCI (Carmel and Martin, 2014; Squair et al., 2021). Moreover, activation of the sensorimotor cortex by excitatory designer receptors exclusively activated only by designer drugs (DREADDs) promotes CS axon growth and increases their projections to the spinal cord (Yang and Martin, 2023). In another study, chemogenetic stimulation of spinal neurons using DREADDs induced axon sprouting and restored locomotion after SCI by administering actuator ligands (Brommer et al., 2021). In addition, such chemogenetic stimulation of CSNs in both supraspinal centers and spinal relay stations results in enhancement of neuronal rewiring and locomotor recovery (Van Steenbergen et al., 2023). Together, neuronal stimulation of the *RhoA*;*Pten*-deleted axons, which have regained the potential to grow and sprout, might be a novel strategy to enhance axon regeneration and motor recovery after SCI.

In this study, we determined whether combining *RhoA*;*Pten* deletion in CSNs with chemogenetic neuronal stimulation synergistically enhances CS circuit rewiring and forelimb functional recovery after cervical SCI in mice. Our results demonstrate that this combinatorial intervention spurred CS axon regrowth. Additionally, such intervention achieved partial restoration of forelimb motor recovery. Our results, therefore, strongly suggest that combinatorial treatment strategies, specifically tailored to the needs of individual patients, will maximize motor recovery in those impacted by traumatic SCI.

## Materials and Methods

### Animals

*RhoA^f/f^* (Chauhan et al., 2011; Katayama et al., 2011; Melendez et al., 2011) and *Pten^f/f^* (The Jackson laboratory; Lesche et al., 2002) mice were crossed to create *RhoA^f/f^*;*Pten^f/f^* mice that were maintained on a C57BL/6 background. Males and females were used in the experiments. Mice were isolated in individual cages in a pathogen-free environment under a 12 h light/dark cycle and fed commercial pellets and water *ad libitum*.

### Ethics declaration

Procedures were performed in accordance with protocols approved by the Weill Cornell Medicine Institutional Animal Care and Use Committee. All experiments were performed in a manner that minimized pain and discomfort of the mice.

### Adeno-associated viruses (AAVs)

The following AAVs were used in experiments: AAV8-Ef1a-fDIO-Cre (AAV8-fDIO-Cre; 2.1 × 10^13^ GC/ml, Addgene, 121675-AAV8; Schneeberger et al., 2019), AAV8-Ef1a-fDIO-mCherry (AAV8-fDIO-mCherry; 1.8 × 10^13^ GC/ml, Addgene, 114471-AAV8), AAV8-CMV-LacZ (AAV8-LacZ; 1.7 × 10^13^ GC/ml, Addgene, 105531-AAV8), AAV1-hsyn-Cre (AAV1-Cre; 1.9 × 10^13^ GC/ml, Addgene, 105553-AAV1), AAV1-hsyn-GFP (AAV1-GFP; 1.0 × 10^12^ GC/ml, Addgene, 50465-AAV1), AAVretro-Ef1a-Flpo (AAVretro-Flpo; 1.6 × 10^13^ GC/ml, Addgene, 55637-AAVrg; Fenno et al., 2014), and AAVretro-hsyn-DIO-hM3Dq-mCherry (AAVretro-DIO-hM3Dq-mCherry; 1.9 × 10^13^ GC/ml, Addgene, 44361-AAVrg; Krashes et al., 2011).

### Surgeries

#### AAV injections

Mice were anesthetized with isoflurane and placed in a stereotaxic frame (Stoelting, 51730D). For brain injections, the scalp was incised (incision size of 10 × 10 mm^2^), and holes were made in the skull at the corresponding AAV injection sites using a round stainless-steel drill (⌀ 0.5 mm). AAVs were injected into the rostral forelimb and caudal forelimb areas (RFA and CFA, respectively) of the sensorimotor cortex [depth of 0.5 mm; coordinates, 1.8 mm anterior, 1.2 mm lateral to the bregma, 0.6 mm posterior, 1.8 mm lateral to the bregma (Wang et al., 2017), 0.2 µl/virus/site] using a Nanoject III injector (Drummond Scientific Company, 3-000-207) tipped with a glass micropipette. For spinal cord AAV injections, skin and muscles on the cervical region of the neck were incised, exposing the C4 vertebrae. AAVs were injected into the C5 region of the spinal cord (depth of 0.5 mm and 1.0 mm; coordinates, 0.5 mm anterior, 0.5 mm lateral to the center of C4 vertebrae, 0.5 mm posterior, 0.5 mm lateral to the center of C4 vertebrae, 0.15 µl/virus/site) using the Nanoject III instrument.

#### Spinal cord injury (SCI)

Dorsal column lesions were made as previously described (Hollis et al., 2016). Briefly, after anesthetization with isoflurane, a laminectomy at the C4 vertebrae was performed to expose the spinal cord. A dorsal column lesion (depth of 1.0 mm) was made at the C5 vertebrae using Vannas spring scissors (2.5 mm cutting edge) to sever the dorsal CST.

#### Analgesia

Prior to skin incision, 100 μl of a mixture of 2% lidocaine and 0.5% bupivacaine was administered subcutaneously as a local analgesic. Buprenorphine (0.5 mg/kg) was administered subcutaneously as a postoperative analgesic immediately following surgery and twice per day thereafter for 3 d. Meloxicam (0.2 mg/kg) was additionally administered subcutaneously following AAV injections. Syringes equipped with 30 G needles were used for all injections.

### Western blots

Dissected brains were sectioned using a mouse coronal brain slicer (Kent Scientific, rbma-200c). Areas of the brain in which GFP fluorescence was detected were collected and homogenized in lysis buffer [50 mM Tris-HCl, pH 8.0, containing 150 mM NaCl, 1% NP-40, 0.5% sodium deoxycholate, 0.1% SDS, and protease inhibitor cocktail (Abcam, ab271306)]. After centrifugation at 10,000 × *g* for 10 min at 4°C, the protein concentration was adjusted to 1 mg/ml. Proteins were separated by SDS-PAGE and transferred to a PVDF membrane (Bio-Rad, 162-0177). Chameleon NIR Duo-Prestained Protein Ladder (LICORbio, 928-60000) was used to estimate protein band sizes. The PVDF membrane was blocked with 5% skim milk in PBS containing 0.05% Tween 20 and then incubated overnight at 4°C with either rabbit anti-RhoA (1:1,000, Cell Signaling Technology, 67B9), rabbit anti-Pten (1:1,000, Cell Signaling Technology, 9188S), or mouse Tuj1 (1:1,000, BioLegend, MMS-435P). After washing, the membrane was incubated with anti-rabbit IRDye 680RD (LI-COR, 925-68072) and anti-mouse IRDye 800 (LI-COR, 925-32213). An Odyssey Clx Imager (LI-COR) was used to detect and quantify antibody-bound proteins.

### Histology

Dissected brains and spinal cords were postfixed in 4% PFA overnight. The tissues were then cryopreserved at 4°C in 30% sucrose/PBS overnight and then embedded in a Tissue-Tek optimal cutting temperature compound (Sakura Finetek). Embedded samples were sliced using a cryostat into 50-µm-thick sections and floated on PBS. The sections were then blocked with 1% bovine serum albumin/PBS for 1 h before incubating at 4°C overnight with rat anti-glial fibrillary acidic protein (GFAP; Thermo Fisher Scientific, 13-0300), rabbit anti-cFos (Cell Signaling Technology, 2250S), rabbit anti-PKCγ (Abcam, ab71558), or rat anti-myelin basic protein (MBP; Abcam, ab7349) antibodies. After washing with 0.1% Tween 20/PBS (PBS-t), the sections were incubated with Alexa Fluor 488 anti-rat (Invitrogen, A21208), Alexa Fluor 488 anti-rabbit IgG antibodies (Invitrogen, A21206), or Alexa Fluor 647 anti-rabbit IgG antibodies (Invitrogen, A31573). The sections were then washed with PBS and mounted onto glass slides (Fisherbrand, 1255015). Sections were counterstained with DAPI (1 µg/ml, Invitrogen, D1306) and washed with PBS-t, and 200 µl of mounting media (VectorLabs, H10000-10) was applied directly onto the sections before covering with coverslips (VWR, 48393-251). The slide-mounted sections were scanned using a confocal microscope (Nikon A1R HD25).

### Quantification of activated CSNs

To quantify cFos expression as a marker of neural activity in CSNs (Fig. 3), coronal sections of the brain that exhibited mCherry^+^ CSNs were selected (5–7 sections per sample). Using ImageJ software, mCherry^+^ CSNs were detected and counted in the confocal 16 bit red channel image. The intensity of cFos (fluorescently stained with Alexa Fluor 488) within the mCherry^+^ CSNs was then detected in the 16 bit green channel image and the cFos^+^ neurons were counted. The percentage of cFos^+^ CSNs was calculated by dividing the cFos^+^/mCherry^+^ CSNs by the total number of mCherry^+^ CSNs.

### Quantification of CST dieback

CST dieback analysis was carried out following established quantification methods with some modifications (Nakamura et al., 2021). To quantify the dieback and regeneration of CST axons, 3–5 sagittal sections of the spinal cord that included the mCherry^+^ CST axons were chosen in each sample, and the mCherry intensities within the CST fibers in the dorsal column were measured by ImageJ software. The center of the lesion was set as 0 µm, and measurements were taken at 100 µm intervals along the rostral–caudal axis (Extended Data Fig. 4-1). Each region of interest was set to a width of 100 µm and a height of 500 µm. To normalize the differences between individual animals, the mCherry intensity in each distance bin was divided by the intensity of mCherry in the rostral 1,000 µm bin (−1,000 µm from the lesion), and this value was defined as the axon index. To assess the suppression of axon dieback relative to control mice, the mCherry axon indices of the *RhoA*;*Pten* double conditional KO (dcKO) mice and the dcKO with DREADD (dcKO^hM3Dq and water^ and dcKO^hM3Dq and DCZ^) mice were divided by the mean index of the control mice (LacZ and LacZ^DCZ^), and this calculated value was defined as the mCherry axon index ratio.

### Quantification of axon collateral projections

Axon collateral projection analysis was carried out following established quantification methods with some modifications (Nakamura et al., 2021). Transverse sections of the spinal cord at 0, 50, 100, 150, and 200 µm rostral, and 50 and 100 µm caudal, to the lesion were chosen to quantify axon collaterals (Fig. 6). To determine the center of the lesion, consecutive sections that likely included the lesion were first identified and the middlemost section was set as the center. The mCherry intensity of CST fibers in the gray matter was measured in each section by ImageJ software. As in prior experiments, the mCherry intensity in the dorsal funiculus at 1,000 µm rostral to the lesion was used for normalization, since axon dieback is limited at this distance and reflects the full transduction of CST neurons. This normalized value was defined as the mCherry axon collateral index (Fig. 6*c,d*).

### Quantification of presynaptic boutons

The total number of mCherry^+^ boutons was quantified by Imaris AI microscopy image analysis software. Boutons with diameters greater than 5 µm were selected and counted from transverse sections of the spinal cord at 0, 50, 100, 150, and 200 µm rostral, and 50 and 100 µm caudal, to the lesion site. The number of boutons was normalized by dividing by the mCherry intensity in the dorsal funiculus 1,000 µm rostral to the lesion. This normalized value was defined as the bouton index (Fig. 6*f*).

### Behavioral analyses

A grid-walking test using an elevated square wire grid (20 × 20 cm^2^ with 1.2 × 1.2 cm^2^ grid cells) was used to evaluate the effects of SCI on the skilled behaviors of treated and control mice. A custom-made transparent acrylic tube (25 cm diameter) was placed over the grid to prevent mice from walking on the grid's outer edges. Limb slips were detected during playbacks of video recordings (30 frames/s) of traversal attempts. A mirror was placed under the grid at an angle of 45°, and the reflection was recorded with another video camera to identify the limbs that slipped from the grid.

Mice were subjected to the grid-walking test prior to SCI (preinjury) and then again at 7, 14, 21, 28, 35, 42, and 49 d postinjury (DPI). Mice were allowed to walk on the grid for 3 min, and foot-slips of the left and right forelimbs were counted during the first 50 forelimb steps. A slip was scored when the forepaw completely missed the grid and the limb fell between the wires, or when the forepaw was correctly placed on the grid but slipped off during the weight-bearing phase (Chao et al., 2012). The percentage of slips was calculated as the number of slips divided by the first 50 steps per trial × 100. To normalize the differences between animals, the slip rate at each DPI was divided by the preinjury rate, and this value was defined as the slip rate index. To show performance deterioration relative to controls, the slip rate indices of mice with *RhoA*;*Pten* deletion in CSNs (dcKO) and dcKO with DREADD mice (dcKO^hM3Dq and water^ and dcKO^hM3Dq and DCZ^) were divided by the mean index of the control mice (LacZ and LacZ^DCZ^), and this value was defined as the slip rate index ratio.

### Evaluation and statistical analyses

Mice were randomly chosen from a *RhoA^f/f^*;*Pten^f/f^* mouse population and divided into cohorts for AAV injections prior to SCI. The mice were then randomly assigned numbers for the grid-walking task, which was conducted in a blinded manner.

Statistical analyses were performed using R software with all quantitative data represented as the standard error of the mean. Differences among groups were statistically analyzed using a two-tailed unpaired *t* test, Wilcoxon rank sum exact test, two-way ANOVA followed by Tukey's test, or Kruskal–Wallis test followed by Wilcoxon rank sum exact test. A *p*-value of <0.05 was considered statistically significant.

### Experimental design

**Figure 1.**
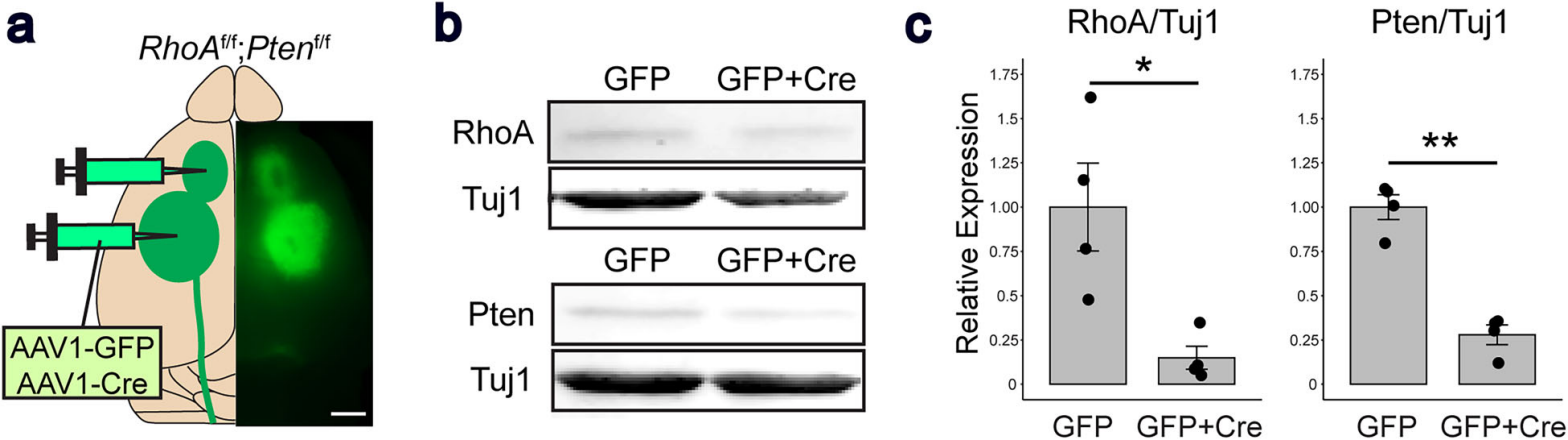
Genetic deletion of *RhoA* and *Pten* in the sensorimotor cortex. ***a***, Schematic illustration showing the AAV1-Cre-mediated gene deletion strategy. AAV1-GFP and AAV1-Cre (GFP + Cre) were injected into the RFA and CFA, respectively, in the brains of *RhoA^f/f^*;*Pten^f/f^* mice. The stereomicroscopic image shows GFP expression in the cerebral cortex (right panel). Scale bar, 2 mm. ***b***, ***c***, Western blot analysis of RhoA (21 kDa) and Pten (54 kDa) in the cerebral cortex of AAV-injected *RhoA^f/f^*;*Pten^f/f^* mice. Tuj1 (55 kDa) was used as a loading control. The relative band intensities of RhoA and Pten, normalized to Tuj1, were significantly reduced in the GFP + Cre coinjected mice. ***c***, Full Western blot images are shown in Extended Data Figure 1-1. Unpaired *t* test (GFP; *n* = 4, GFP + Cre; *n* = 4). *p < 0.05, **p < 0.005. Specific data is in Table 1.

To delete *RhoA* and *Pten* in CSNs, 6-week-old *RhoA^f/f^;Pten^f/f^* mice were injected with AAV1-GFP or AAV1-Cre in the RFA and CFA of the sensorimotor cortex (Fig. 1*a*). Two weeks later, animals were killed by CO_2_ exposure, and brains were dissected and processed for Western blotting.

10.1523/ENEURO.0359-24.2025.f1-1 Figure 1-1 Genetic deletion of *RhoA* and *Pten* in the sensorimotor cortex. (a) Raw western blot image of RhoA (21  kDa) and Tuj1 (55  kDa) in the cerebral cortex of AAV-injected *RhoA^f/f^*;*Pten^f/f^* mice. (b) Green channel image isolated from the raw image (a). (c) Red channel image isolated from the raw image (a). (d) Raw western blot image of Pten (54  kDa) and Tuj1 (55  kDa) in the cerebral cortex of AAV-injected *RhoA^f/f^*;*Pten^f/f^* mice. (e) Green channel image isolated from the raw image (d). (f) Red channel image isolated from the raw image (d). Dotted boxes are the indicated bands for Figure 1. Download Figure 1-1, TIF file.

Dorsal column transections were performed to completely sever the CSTs of mice (Extended Data Fig. 2-2). The effectiveness of the procedure was assessed by euthanizing *RhoA^f/f^*;*Pten^f/f^* mice 2 weeks after transection and examining their spinal cord tissues after immunohistochemical (IHC) processing. MBP antibodies were used to evaluate whether the dorsal column had been fully severed, and protein kinase C gamma (PKCγ) antibodies, which specifically bind to active CSNs, were used to evaluate whether the CST was transected by the lesion.

**Figure 2.**
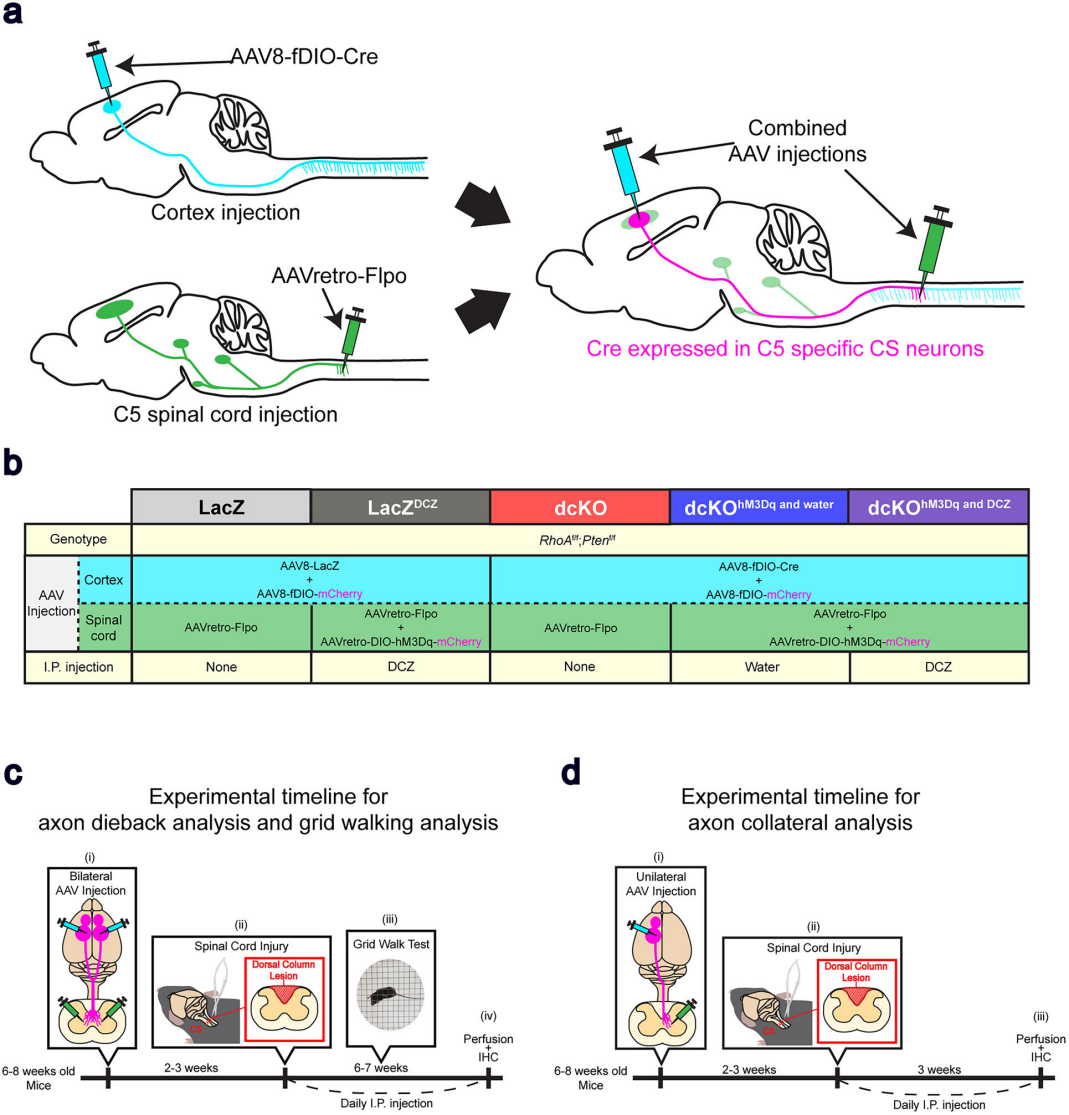
Experimental design. ***a***, Schematic of combinatorial AAV injections. Cortical injections of AAV8-fDIO-Cre anterogradely infects CSNs, while spinal cord injections of AAVretro-Flpo retrogradely infects multiple spinal neurons. Combination of the two injections induces the expression of Cre in C5 spinal cord-specific CSNs. Representative raw images of mCherry fluorescence are shown in Extended Data Figure 2-1. ***b***, AAV injection strategy: To delete *RhoA* and *Pten* in CSNs, AAVretro-Flpo was injected into the C5 spinal cord of *RhoA^f/f^*;*Pten^f/f^* mice, followed by injection of AAV8-fDIO-Cre into the cortex. To express the excitatory DREADD receptor, hM3Dq in CSNs, AAVretro-DIO-hM3Dq-mCherry was injected into the spinal cord. To visualize CSNs, AAV8-fDIO-mCherry was also injected into the cortex of each mouse group. In LacZ and LacZ^DCZ^ cohorts, AAV8-LacZ was injected instead of AAV8-fDIO-Cre, which will result in failure of dcKO and hM3Dq expression. Intraperitoneal (i.p.) injections of DCZ were performed in LacZ^DCZ^ and dcKO^hM3Dq and DCZ^ mice, and i.p. injections of water were performed in dcKO^hM3Dq and water^ mice. ***c***, Experimental schematic and timeline for axon dieback and grid-walking analyses: (1) AAVs were bilaterally injected into the sensorimotor cortex and the C5 level of the spinal cord in 6–8-week-old *RhoA^f/f^*;*Pten^f/f^* mice. Representative images of dorsal column lesion are shown in Extended Data Figure 2-2. (2) A C5 dorsal column lesion was performed 2–3 weeks after AAV injections. (3) The grid-walking test was performed weekly thereafter, followed by perfusion at 6–7 weeks postinjury. (4) Daily i.p. injections were performed during the postinjury period, and the brain and spinal cord tissues were processed for IHC analyses. ***d***, Experimental schematic and timeline for axon collateral analysis: (1) AAVs were unilaterally injected into the cortex and the C5 level of the spinal cord in 6–8-week-old *RhoA^f/f^*;*Pten^f/f^* mice. (2) A C5 dorsal column lesion was performed 2–3 weeks after AAV injections. (3) Daily i.p. injections were performed during the postinjury period, followed by perfusion and IHC analyses of brains and spinal cords at 3 weeks postinjury.

To assess the levels of axon dieback and the impacts on forelimb motor behavior following *RhoA;Pten* deletion and neuronal activation using excitatory DREADDs, 6–8-week-old *RhoA^f/f^;Pten^f/f^* mice were injected with various combinations of AAVs. Intracortical injections of AAV8 infects all or most of the forelimb-related CST tissues anterogradely, while injection of AAVretro into the C5 spinal cord infects the CST and other descending spinal tracts retrogradely (Fig. 2*a*). By combining the cortical injection of an AAV8 vector encoding a recombinase-dependent gene of interest, with a C5 spinal cord injection of an AAVretro vector providing the needed recombinase, we can express the gene of interest in CSNs projecting to the spinal cord including the C5 level (Fig. 2*a*).

10.1523/ENEURO.0359-24.2025.f2-1 Figure 2-1 **Stereo microscope image of brain and spinal cord after perfusion.** (a-b) Raw stereo microscope image of the brain (a) and spinal cord (b) after perfusion in a dcKO^hM3Dq and DCZ^ mouse. C5 spinal cord-specific CS neurons are visualized by mCherry fluorescence (red channel image on right). The mCherry signal is disrupted by the C5 dorsal column lesion (b). Scale bar, 1  mm. Download Figure 2-1, TIF file.

10.1523/ENEURO.0359-24.2025.f2-2 Figure 2-2 **Confocal images of complete dorsal column lesion.** (a) Merged images of PKCγ (red), MBP (green), and DAPI (blue) of spinal cords at the lesion site (0  µm), and -1000  µm (rostral) and 300  µm (caudal) to the lesion. (b-d) Individual images of PKCγ (b), MBP (c), and DAPI (d) from the merged image (a). PKCγ in the dorsal column shows the active corticospinal neurons, which fade at the lesion and caudal to the lesion (b). MBP reveals the completeness of the dorsal column lesion with minimal damage to other nervous tissue (c). Scale bar, 500  µm. Download Figure 2-2, TIF file.

Five mouse cohorts were prepared for this study (Fig. 2*b*):

1. LacZ
  AAV8-LacZ and AAV8-fDIO-mCherry were injected into the sensorimotor cortex, while AAVretro-Flpo was injected into the C5 spinal cord of *RhoA^f/f^;Pten^f/f^* mice. In this cohort, mCherry will be expressed in CSNs projecting to the C5 spinal cord.
2. LacZ^DCZ^
  AAV8-LacZ and AAV8-fDIO-mCherry were injected into the sensorimotor cortex, while AAVretro-Flpo and AAV8-DIO-hM3Dq-mCherry were injected into the cervical spinal cord of *RhoA^f/f^;Pten^f/f^* mice. This cohort will express mCherry in CSNs projecting to the C5 spinal cord. The DREADD agonist, deschloroclozapine (DCZ; MCE, HY-42110), was intraperitoneally (i.p.) injected after the SCI.
3. Double conditional knock out (dcKO)
  AAV8-fDIO-Cre and AAV8-fDIO-mCherry were injected into the sensorimotor cortex, and AAVretro-Flpo was injected into the cervical spinal cord of *RhoA^f/f^;Pten^f/f^* mice. In this cohort, *RhoA*;*Pten* are deleted and mCherry will be expressed in CSNs projecting to the C5 spinal cord.
4. Double conditional knock out with hM3Dq with administration of water (dcKO^hM3Dq and water^)
  AAV8-fDIO-Cre and AAV8-fDIO-mCherry were injected into the sensorimotor cortex, and AAVretro-Flpo and AAV8-DIO-hM3Dq-mCherry were injected into the cervical spinal cords of *RhoA^f/f^;Pten^f/f^* mice. *RhoA*;*Pten* are deleted in this cohort, and hM3Dq and mCherry will be expressed in CSNs projecting to the C5 spinal cord. Water was i.p. injected after the SCI.
5. Double conditional knock out with hM3Dq with administration of DCZ (dcKO^hM3Dq and DCZ^)
  AAV8-fDIO-Cre and AAV8-fDIO-mCherry were injected into the sensorimotor cortex, and AAVretro-Flpo and AAV8-DIO-hM3Dq-mCherry were injected into the cervical spinal cords of *RhoA^f/f^;Pten^f/f^* mice. In this cohort, *RhoA*;*Pten* are deleted, and hM3Dq and mCherry will be expressed in CSNs projecting to the C5 spinal cord. DCZ was i.p. injected after SCI.

The LacZ and LacZ^DCZ^ groups were prepared as control groups in which *RhoA*;*Pten* are not deleted and excitatory DREADD is not expressed in the CST due to the lack of Cre (Fig. 2*b*). The dcKO group only expresses the conditional KO (Fig. 2*b*). The dcKO^hM3Dq and water^ and dcKO^hM3Dq and DCZ^ groups both express the conditional KO with excitatory DREADD, but the dcKO^hM3Dq and water^ receives i.p. injections of water via a 30 G syringe which does not activate the DREADD receptor (Fig. 2*b*).

Two to three weeks after bilateral AAV injections, lesions were made in the C5 spinal cord, and mice were subjected to weekly grid-walking tests starting 1 week post-SCI (Fig. 2*c*). Starting the day after injury, DCZ was i.p. injected with a 30 G syringe into LacZ^DCZ^ and dcKO^hM3Dq and DCZ^ mice twice a day at a concentration of 100 µg/kg (Nagai et al., 2020; Nentwig et al., 2022). Injections continued until 42 DPI. On the days when mice were subjected to their weekly grid-walking tests, DCZ was administered twice daily after each test. To confirm whether hM3Dq was affecting behavior in the hM3Dq-expressing mice, an additional week of grid-walking tests was performed without DCZ. After the final grid-walking test, the LacZ mice and dcKO mice were transcardially perfused with 4% PFA/PBS, while the LacZ^DCZ^ mice, dcKO^hM3Dq and water^ mice, and dcKO^hM3Dq and DCZ^ mice were administered with DCZ and perfused 2–3 h later (Fig. 2*c*). The brains and spinal cords were then dissected and processed for immunohistochemistry.

To quantify axon collateral projections, 6–8-week-old *RhoA^f/f^;Pten^f/f^* mice were injected with AAVs unilaterally according to the injection plans to establish the LacZ^DCZ^, dcKO, and dcKO^hM3Dq and DCZ^ cohorts (Fig. 2*d*). SCIs were made in the C5 spinal cord 2 weeks after AAV injections. Mice were then perfused at 21 DPI, and brains and spinal cords were dissected and processed for IHC analyses (Fig. 2*d*).

## Results

A recent study showed that modulation of extrinsic and intrinsic signaling pathways through deletion of both *RhoA* and *Pten* suppresses axon dieback after thoracic SCI (Nakamura et al., 2021). Excitation of CSNs has also been shown to promote axon sprouting following CST injury (Carmel and Martin, 2014). We examined whether combining these two approaches would augment motor recovery after cervical SCI by deleting *RhoA* and *Pten* in CSNs and chemogenetically activating those neurons in mice. We assessed the anatomical and functional effects of this combinatorial approach on CS circuit recovery after cervical SCI.

### Cre recombinase induces sufficient *RhoA*;*Pten* deletion in the sensorimotor cortex

We first determined the effects of genetic deletion of *RhoA* and *Pten* in CSNs by injecting AAV-Cre into the sensorimotor cortex of *RhoA^f/f^;Pten^f/f^* mice (Fig. 1). Coinjection of AAV1-GFP and AAV1-Cre (GFP + Cre mice, *n* = 4) into the sensorimotor cortex lowered the expression of both RhoA and Pten proteins compared with control mice (GFP only, *n* = 4; Fig. 1*b*). Quantitative analysis also showed significantly lower protein levels in GFP + Cre mice, indicating that Cre expression induced sufficient genetic codeletion of *RhoA* and *Pten* (Fig. 1*c*, Table 1).

**Table 1.** Western blot analysis statistical data

	95% confidence interval	*p*-value
RhoA/Tuj1	CI^low^[0.5024474] CI^up^[0.9392495]	0.0161
Pten/Tuj1	CI^low^[0.2227772] CI^up^[1.4780436]	0.0002

Table summarizing the statistical analysis of relative expression in Western blotting. Normal distribution, unpaired *t* test.

### Combination of *RhoA;Pten* deletion and neuronal activation suppresses axon dieback after SCI

To delete *RhoA* and *Pten* specifically in CSNs, AAV8-fDIO-Cre was injected into the sensorimotor cortex, and AAVretro-Flpo was injected into the cervical spinal cord of *RhoA^f/f^;Pten^f/f^* mice (hereafter referred to as dcKO mice; Fig. 2).

**Figure 3.**
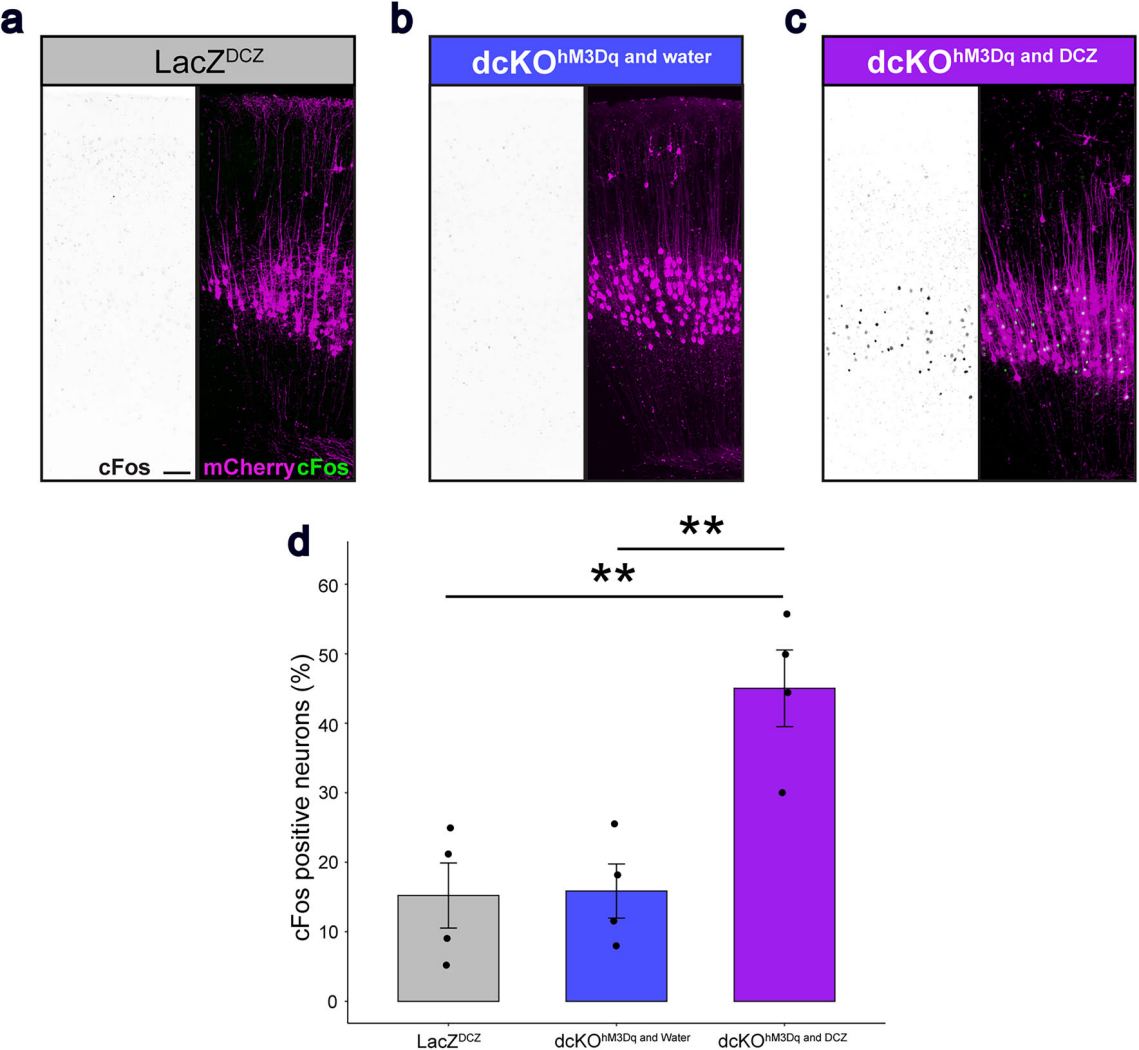
Neuronal stimulation by excitatory DREADDs in the sensorimotor cortex. ***a–c***, Representative images of cFos staining (black on the left, green on the right) and mCherry (magenta on the right) in (***a***) LacZ^DCZ^ (*n* = 4), (***b***) dcKO^hM3Dq and water^ (*n* = 5), and (***c***) dcKO^hM3Dq and DCZ^ (*n* = 4) mice. Scale bar, 100 µm. ***d***, Quantitative analysis of cFos^+^ neurons in mCherry^+^ CSNs in LacZ^DCZ^, dcKO^hM3Dq and water^, and dcKO^hM3Dq and DCZ^ mice. Two-way ANOVA followed by Tukey's test. **p < 0.005. Specific data is in Table 2.

Activation of the DREADD receptor, hM3Dq, by the addition of DCZ was evaluated by examining cFos expression in LacZ^DCZ^ (*n* = 4), dcKO^hM3Dq and water^ (*n* = 5), and dcKO^hM3Dq and DCZ^ (*n* = 4) mice (Fig. 3). The number of cFos^+^ and mCherry^+^ CSNs in dcKO^hM3Dq and DCZ^ mice was significantly higher than the other groups, indicating that Cre successfully induced the expression of the excitatory hM3Dq receptor and DCZ functioned as an actuator ligand (Fig. 3*c*, Table 2).

**Table 2.** cFos analysis statistical data

	95% confidence interval	*p*-value
Two-way ANOVA		0.0023
LacZ^DCZ^ versus dcKO^hM3Dq and water^	CI^low^[−18.09907] CI^up^[19.3758]	0.9950
LacZ^DCZ^ versus dcKO^hM3Dq and DCZ^	CI^low^[11.08059] CI^up^[48.55546]	0.0041
dcKO^hM3Dq and water^ versus dcKO^hM3Dq and DCZ^	CI^low^[−47.91709] CI^up^[−10.44223]	0.0047

Table summarizing the statistical analysis of cFos positive neuron percentage. Normal distribution and two-way ANOVA followed by Tukey's test.

**Figure 4.**
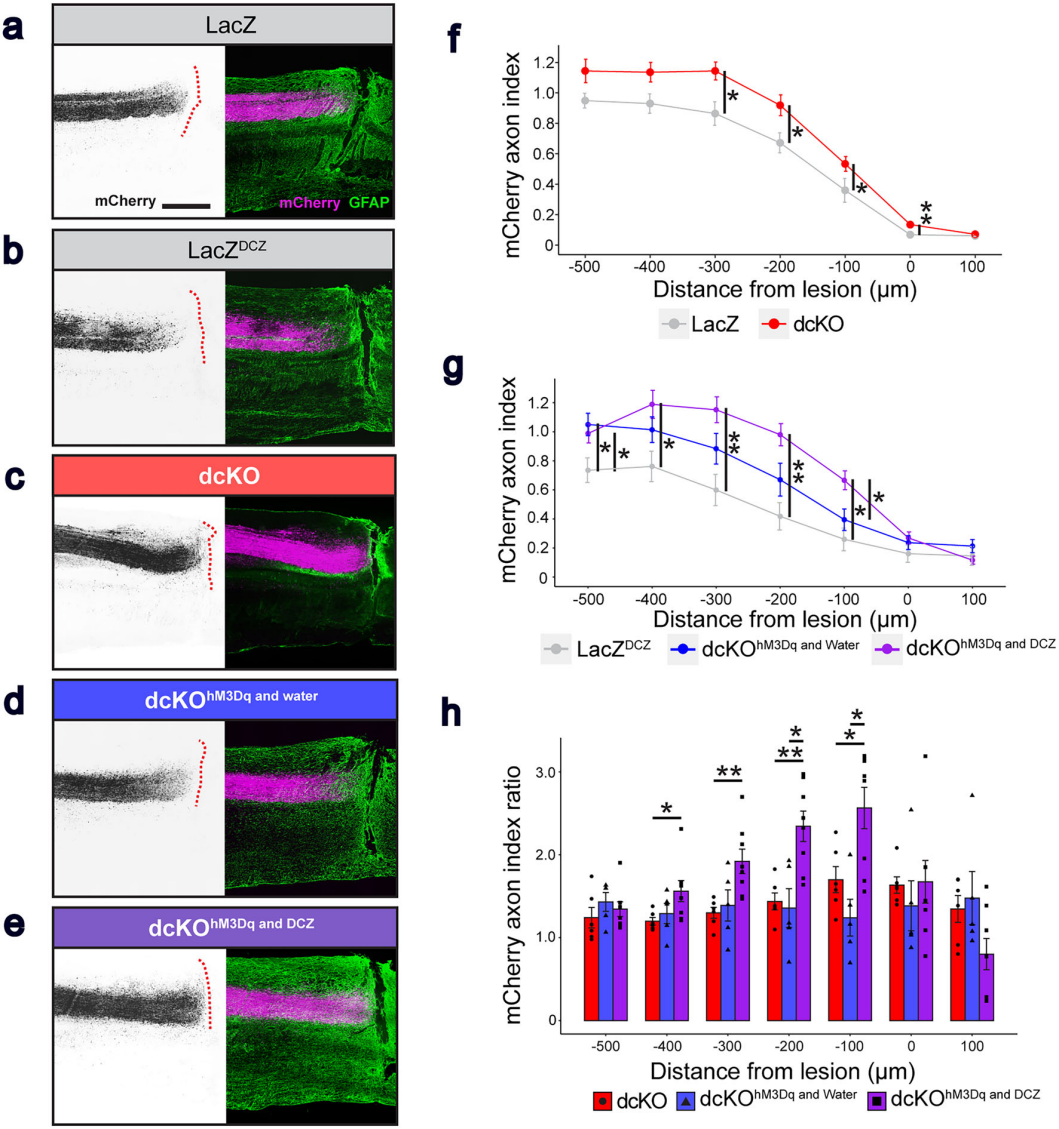
*RhoA*;*Pten* dcKO mice with DREADDs show suppression of axon dieback in the CST after SCI. ***a–e***, Representative images of mCherry^+^ CST axons (black on the left, magenta on the right) and GFAP^+^ tissues (green on the right) in the cervical spinal cord in (***a***) LacZ (*n* = 6), (***b***) LacZ^DCZ^ (*n* = 6), (***c***) dcKO (*n* = 7), (***d***) dcKO^hM3Dq and water^ (*n* = 6), and (***e***) dcKO^hM3Dq and DCZ^ (*n* = 8) mice at 42 DPI (***a***, ***c***) and 49 DPI (***b***, ***d***, ***e***). Dotted red lines indicate the borders of the GFAP^+^ glial scars and the GFAP^−^ fibrotic scars. Scale bar, 500 μm. ***f***, Quantification of CST axons in the dorsal funiculus in LacZ and dcKO mice. Wilcoxon rank sum exact test. ***g***, Quantification of CST axons in the dorsal funiculus in LacZ^DCZ^, dcKO^hM3Dq and water^, and dcKO^hM3Dq and DCZ^ mice. Kruskal–Wallis test followed by Wilcoxon rank sum exact test. ***h***, Quantification of CST axon ratios in the dorsal funiculus of dcKO and dcKO^hM3Dq and DCZ^ mice. Regions of interest are identified according to Extended Data Figure 4-1. Kruskal–Wallis test followed by Wilcoxon rank sum exact test. *p < 0.05, **p < 0.005. Specific data is in Table 3.

Sagittal sections of the spinal cord were examined to evaluate CS axon lengths (Fig. 4). Signal intensities of the astrocyte marker, GFAP, were elevated near the transection sites in all the groups, indicating that glial scars had formed near the lesions. In LacZ (*n* = 7) and LacZ^DCZ^ (*n* = 6) mice, >50% of the mCherry^+^ axons were present at 200–500 µm rostral to the lesion in the dorsal funiculus, indicating that axon dieback had occurred (Fig. 4*a,c*). In contrast, >50% of axons were observed at 100 µm rostral to the lesion in dcKO (*n* = 7), dcKO^hM3Dq and water^ (*n* = 5), and dcKO^hM3Dq and DCZ^ (*n* = 8) mice (Fig. 4*b,d*). The CST axon indices in dcKO, dcKO^hM3Dq and water^, and dcKO^hM3Dq and DCZ^ mice showed reduced axon dieback compared with LacZ and LacZ^DCZ^ mice. To further evaluate the differences between the dcKO, dcKO^hM3Dq and water^, and dcKO^hM3Dq and DCZ^ mice, we measured the improvements in axon dieback suppression compared with the LacZ and LacZ^DCZ^ cohorts. In both dcKO and dcKO^hM3Dq and DCZ^ mice, the CST axon indices were significantly higher at 100–300 µm rostral to the lesion compared with control mice (Fig. 4*f,g*, Table 3a,b). These results suggest that *RhoA*;*Pten* deletion suppresses axon dieback at cervical levels. Moreover, the combination of CSN stimulation via excitatory DREADDs with *RhoA*;*Pten* deletion resulted in greater reductions in axon dieback rostral to the lesion over *RhoA*;*Pten* deletion alone (Fig. 4*h*,Table 3c).

10.1523/ENEURO.0359-24.2025.f4-1 Figure 4-1 **Region of interest in the axon dieback analysis.** (a-e) Representative images of mCherry^+^ CST axons (magenta on the left, black on the right) and GFAP^+^ tissues (green on the left) in the cervical spinal cord in LacZ (a), LacZ^DCZ^ (b), dcKO (c), dcKO^hM3Dq and water^ (d), and dcKO^hM3Dq and DCZ^ (e) mice at 42 DPI (a, c) and 49 DPI (b, d, e). White boxes in immunofluorescence images on the left represent regions of interest for the axon dieback analysis. Scale bar, 500  µm. (f-j) Maximized mCherry^+^ CST axon images of the regions of interest in a-e. Scale bar, 100  µm. Download Figure 4-1, TIF file.

**Table 3.** Axon dieback analysis statistical data

a. Data summary of Figure 4*f*							
Distance from lesion (µm)	−500	−400	−300	−200	−100	0	100
LacZ versus dcKO *p*-value	0.1255	0.0519	0.0303	0.0087	0.0087	0.0043	0.3290
b. Data summary of Figure 4*g*							
Distance from lesion (µm)	−500	−400	−300	−200	−100	0	100
Kruskal–Wallis *p*-value	0.0253	0.0241	0.0123	0.0057	0.0129	0.0948	0.1346
LacZ^DCZ^ versus dcKO^hM3Dq and water^ *p*-value	0.0170	0.1770	0.1255	0.1255	0.1770		
LacZ^DCZ^ versus dcKO^hM3Dq and DCZ^ *p*-value	0.0290	0.0080	0.0047	0.0027	0.0130		
dcKO^hM3Dq and water^ versus dcKO^hM3Dq and DCZ^ *p*-value	0.5240	0.2220	0.1274	0.0451	0.0300		
c. Data summary of Figure 4*h*							
Distance from lesion (µm)	−500	−400	−300	−200	−100	0	100
Kruskal–Wallis *p*-value	0.3658	0.0386	0.0102	0.0073	0.0106	0.3595	0.0825
dcKO versus dcKO^hM3Dq and water^ *p*-value		0.4290	0.7922	0.9307	0.0820		
dcKO versus dcKO^hM3Dq and DCZ^ *p*-value		0.1710	0.0653	0.0186	0.0110		
dcKO^hM3Dq and water^ versus dcKO^hM3Dq and DCZ^ *p*-value		0.0130	0.0013	0.0027	0.0430		

Table summarizing the statistical analysis of mCherry axon index and the ratio. Non-normal distribution and Wilcoxon rank sum exact test (a). Non-normal distribution and Kruskal–Wallis test followed by Wilcoxon rank sum exact test (b). Non-normal distribution and Kruskal–Wallis test followed by Wilcoxon rank sum exact test (c).

### Combination of *RhoA*;*Pten* deletion and excitatory DREADDs promotes forelimb motor recovery

**Figure 5.**
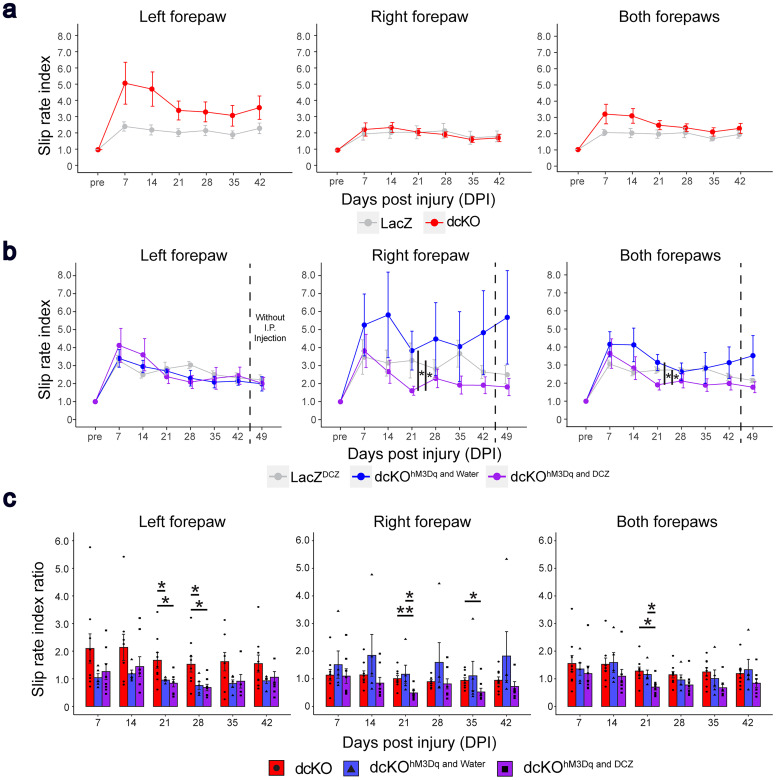
The combination of *RhoA*;*Pten* deletion and neuronal stimulation enhances motor recovery after SCI. ***a***, Forelimb slip rate indices of the left (left panels), right (middle panels), and both forepaws (right panels) in LacZ (*n* = 7) and dcKO mice (*n* = 9) on the grid-walking test. Wilcoxon rank sum exact test. ***b***, Forelimb slip rate indices of the left (left panels), right (middle panels), and both forepaws (right panels) in LacZ^DCZ^ (*n* = 7), dcKO^hM3Dq and water^ (*n* = 6), and dcKO^hM3Dq and DCZ^ mice (*n* = 8) on the grid-walking test. Dotted lines indicate when DCZ administration was stopped. Kruskal–Wallis test followed by Wilcoxon rank sum exact test. ***c***, Slip ratios of the left (left panel), right (middle panel), and both forepaws (right panel) in dcKO, dcKO^hM3Dq and water^, and dcKO^hM3Dq and DCZ^ mice. Kruskal–Wallis test followed by Wilcoxon rank sum exact test. *p < 0.05, **p < 0.005. Specific data is in Table 4.

To determine whether the combination of *RhoA*;*Pten* deletion in CSNs paired with neural activation can enhance motor recovery after SCI, we subjected mice to a grid-walking test (Chao et al., 2012). In this analysis of skilled behaviors, the slip rate index showed no significant differences between LacZ (*n* = 7) and dcKO mice (*n* = 9; Fig. 5*a*, Table 4a). In contrast, the dcKO with DREADD mice (dcKO^hM3Dq and DCZ^; *n* = 8) showed significantly lower slip rates at 21–35 DPI in both forepaws compared with dcKO^hM3Dq and water^ mice (*n* = 5; Fig. 5*b*, Table 4b). Right forepaw slip rate indices (a normalized value where each slip rate is divided by the preinjury slip rate) in the dcKO^hM3Dq and DCZ^ mice were significantly lower than those of the dcKO mice at 21 DPI (Fig. 5*b*, Table 4b). When both forepaws were examined together, the slip rate was significantly lower for dcKO^hM3Dq and DCZ^ mice at 21 DPI compared with LacZ^DCZ^ mice (Fig. 5*b*, Table 4b). To further evaluate the differences between the dcKO, dcKO^hM3Dq and water^, and dcKO^hM3Dq and DCZ^ mice, we measured the deterioration ratio of slip rate by comparing to the LacZ and LacZ^DCZ^ cohorts (Fig. 5*c*, Table 4c). In the left forepaw, dcKO mice at 21 and 28 DPI showed significantly higher ratio. In the right forepaw, dcKO^hM3Dq and DCZ^ mice at 21 and 35 DPI showed significantly lower ratio. In both forepaws, dcKO^hM3Dq and DCZ^ mice at 21 DPI showed significantly lower ratio. These results indicate that *RhoA*;*Pten* deletion in CSNs alone has limited effect on forelimb motor recovery; however, the addition of excitatory DREADDs significantly enhances forelimb motor functioning after SCI.

**Table 4.** Grid-walking analysis statistical data

a. Data summary of Fig.5a								
	Days post injury(DPI)	7	14	21	28	35	42	
Left forepaw	LacZ vs dcKO p-value	0.0800	0.1400	0.1000	0.2400	0.2200	0.2900	
Right forepaw	LacZ vs dcKO p-value	0.7900	0.7900	0.8700	0.7900	0.7100	0.9200	
Both forepaws	LacZ vs dcKO p-value	0.1400	0.0800	0.2900	0.0920	0.2400	0.6300	
b. Data summary of Fig.5b								
	Days post injury(DPI)	7	14	21	28	35	42	49
Left forepaw	Kruskal-Wallis p-value	0.9685	0.6742	0.6694	0.0989	0.3991	0.9560	0.8426
	LacZ^DCZ^ vs dcKO^hM3Dq and Water^ p-value							
	LacZ^DCZ^ vs dcKO^hM3Dq and DCZ^ p-value							
	dcKO^hM3Dq and Water^ vs dcKO^hM3Dq and DCZ^ p-value							
Right forepaw	Kruskal-Wallis p-value	0.2875	0.1986	0.0199	0.4315	0.0652	0.1036	0.0650
	LacZ^DCZ^ vs dcKO^hM3Dq and Water^ p-value			0.4600				
	LacZ^DCZ^ vs dcKO^hM3Dq and DCZ^ p-value			0.0180				
	dcKO^hM3Dq and Water^ vs dcKO^hM3Dq and DCZ^ p-value			0.0370				
Both forepaws	Kruskal-Wallis p-value	0.4619	0.1782	0.0379	0.1115	0.0610	0.3065	0.1475
	LacZ^DCZ^ vs dcKO^hM3Dq and Water^ p-value			0.8070				
	LacZ^DCZ^ vs dcKO^hM3Dq and DCZ^ p-value			0.0450				
	dcKO^hM3Dq and Water^ vs dcKO^hM3Dq and DCZ^ p-value			0.0320				
c. Data summary of Fig.5c								
	Days post injury(DPI)	7	14	21	28	35	42	
Left forepaw	Kruskal-Wallis p-value	0.2061	0.5409	0.0210	0.0196	0.2479	0.4169	
	dcKO vs dcKO^hM3Dq and Water^ p-value			0.0330	0.0450			
	dcKO vs dcKO^hM3Dq and DCZ^ p-value			1.0000	0.6060			
	dcKO^hM3Dq and Water^ vs dcKO^hM3Dq and DCZ^ p-value			0.0180	0.0140			
Right forepaw	Kruskal-Wallis p-value	0.6629	0.1156	0.0049	0.3857	0.0449	0.1659	
	dcKO vs dcKO^hM3Dq and Water^ p-value			0.7888		0.3500		
	dcKO vs dcKO^hM3Dq and DCZ^ p-value			0.0184		0.1400		
	dcKO^hM3Dq and Water^ vs dcKO^hM3Dq and DCZ^ p-value			0.0032		0.0230		
Both forepaws	Kruskal-Wallis p-value	0.2971	0.1050	0.0165	0.0594	0.0620	0.1536	
	dcKO vs dcKO^hM3Dq and Water^ p-value			0.4376				
	dcKO vs dcKO^hM3Dq and DCZ^ p-value			0.0451				
	dcKO^hM3Dq and Water^ vs dcKO^hM3Dq and DCZ^ p-value			0.0079				

Table summarizing the statistical analysis of slip rate index and the ratio. Non-normal distribution and Wilcoxon rank sum exact test (a). Non-normal distribution and Kruskal–Wallis test followed by Wilcoxon rank sum exact test (b). Non-normal distribution and Kruskal–Wallis test followed by Wilcoxon rank sum exact test (c).

### Combination of *RhoA*;*Pten* deletion and excitatory DREADDs promotes formation of presynaptic boutons after SCI

**Figure 6.**
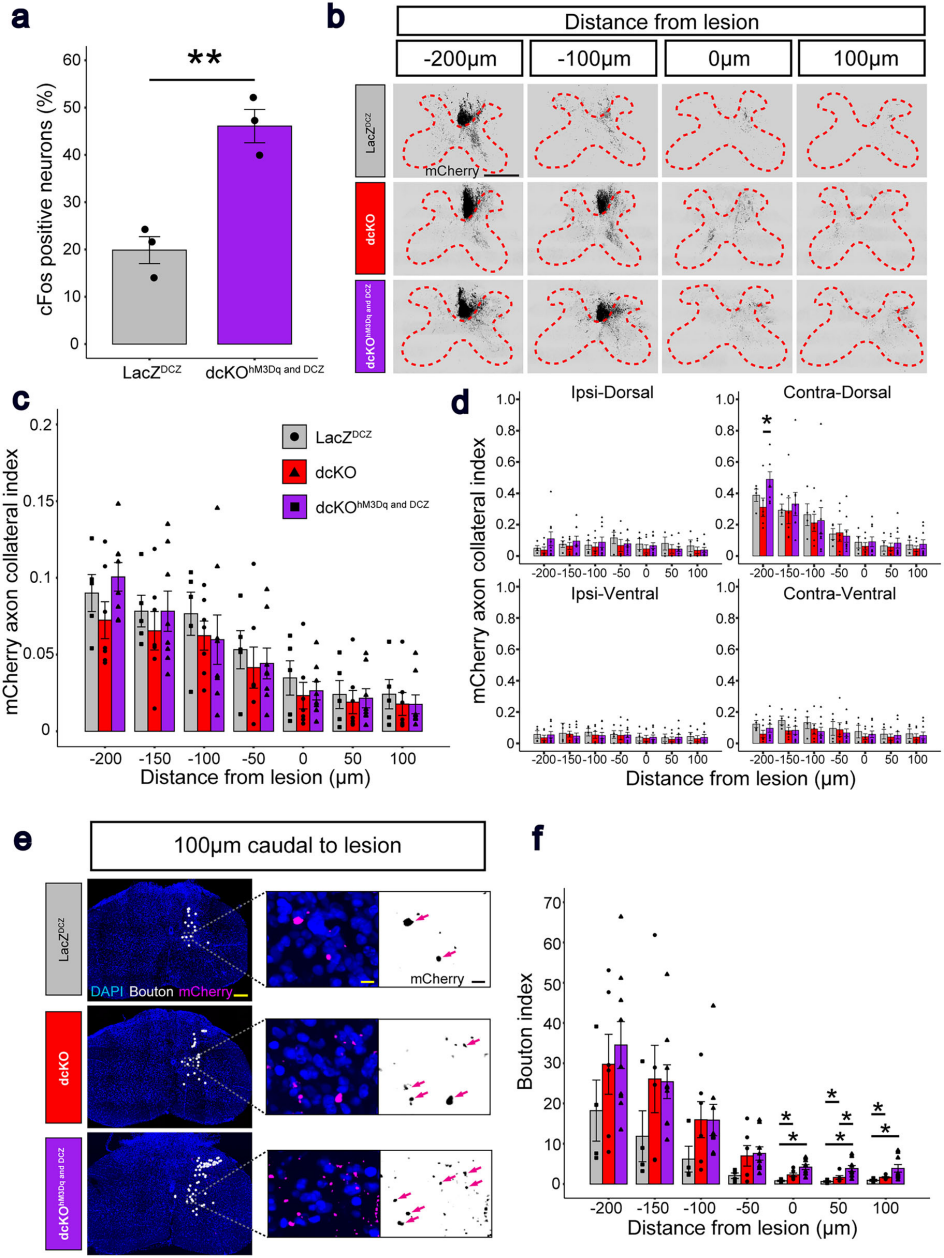
The combination of *RhoA* and *Pten* deletion and neuronal stimulation promotes synaptogenesis in the CST after SCI. ***a***, Quantitative analysis of cFos^+^ neurons in mCherry^+^ CSNs in LacZ^DCZ^ (*n* = 3) and dcKO^hM3Dq and DCZ^ mice (*n* = 3). Unpaired *t* test. ***b***, Representative images of mCherry^+^ CST axons (black) in the cervical spinal cord in LacZ^DCZ^ (top), dcKO (middle), and dcKO^hM3Dq and DCZ^ (bottom) mice at 21 DPI. Dotted red lines indicate the borders between the white and gray matter. Scale bar, 500 μm. ***c***, Quantification of mCherry^+^ CST axon collateral indices in the gray matter. Kruskal–Wallis test followed by Wilcoxon rank sum exact test. ***d***, Quantification of mCherry^+^ CST axon collateral indices in four regions: ipsidorsal (left top), ipsiventral (right bottom), contradorsal (right top), and contraventral (right bottom) in LacZ^DCZ^ (*n* = 4), dcKO (*n* = 4), and dcKO^hM3Dq and DCZ^ mice (*n* = 7). Kruskal–Wallis test followed by Wilcoxon rank sum exact test. ***e***, Representative images of mCherry^+^ (magenta on left and center, black on right), presynaptic boutons (white dots, analyzed by IMARIS on left and center), and DAPI (blue, on left and center) within the gray matter in LazZ^DCZ^ (top row), dcKO (middle row), and dcKO^hM3Dq and DCZ^ (bottom row) mice at 21 DPI. Magenta arrows indicate boutons. Scale bars, 200 μm (left) and 10 μm (right). ***f***, Quantification of boutons in the gray matter in LacZ^DCZ^ (*n* = 4), dcKO (*n* = 4), and dcKO^hM3Dq and DCZ^ mice (*n* = 7). Kruskal–Wallis test followed by Wilcoxon rank sum exact test. *p < 0.05, **p < 0.005. Specific data is in Table 5.

Activation of CSNs by excitatory DREADDs at 21 DPI was evaluated by examining cFos expression in dcKO^hM3Dq and DCZ^ mice (*n* = 3) and their controls (LacZ^DCZ^, *n* = 3; Fig. 6*a*,Table 5a). mCherry^+^ CSNs in dcKO^hM3Dq and DCZ^ mice showed greater cFos expression than CSNs in LacZ^DCZ^ mice, indicating that excitatory DREADDs were induced successfully to activate CSNs.

**Table 5.** Axon collateral and bouton analysis statistical data

a. Data summary of Figure 6*a*								
		95% confidence interval		*p*-value				
	LacZ^DCZ^ versus dcKO^hM3Dq and DCZ^	CI^low^[13.67379]	CI^up^[38.79031]	0.004396				
b. Data summary of Figure 6*c*								
	Days postinjury (DPI)	−200	−150	−100	−50	0	50	100
	Kruskal–Wallis *p*-value	0.5392	0.8333	0.8767	0.9597	0.9155	0.9082	0.9803
	LacZ^DCZ^ versus dcKO *p*-value							
	LacZ^DCZ^ versus dcKO^hM3Dq and DCZ^ *p*-value							
	dcKO versus dcKO^hM3Dq and DCZ^ *p*-value							
c. Data summary of Figure 6*d*								
	Days postinjury (DPI)	−200	−150	−100	−50	0	50	100
Ipsidorsal	Kruskal–Wallis *p*-value	0.1965	0.762	0.3693	0.2158	0.26	0.281	0.2424
	LacZ^DCZ^ versus dcKO *p*-value							
	LacZ^DCZ^ versus dcKO^hM3Dq and DCZ^ *p*-value							
	dcKO versus dcKO^hM3Dq and DCZ^ *p*-value							
Contradorsal	Kruskal–Wallis *p*-value	0.0294	0.8359	0.7621	0.7317	0.5421	0.4992	0.2434
	LacZ^DCZ^ versus dcKO *p*-value	0.2401						
	LacZ^DCZ^ versus dcKO^hM3Dq and DCZ^ *p*-value	0.3159						
	dcKO versus dcKO^hM3Dq and DCZ^ *p*-value	0.0292						
Ipsiventral	Kruskal–Wallis *p*-value	0.2063	0.4207	0.5025	0.541	0.179	0.1933	0.2404
	LacZ^DCZ^ versus dcKO *p*-value							
	LacZ^DCZ^ versus dcKO^hM3Dq and DCZ^ *p*-value							
	dcKO versus dcKO^hM3Dq and DCZ^ *p*-value							
Contraventral	Kruskal–Wallis *p*-value	0.2763	0.3678	0.4795	0.7963	0.3071	0.4042	0.3393
	LacZ^DCZ^ versus dcKO *p*-value							
	LacZ^DCZ^ versus dcKO^hM3Dq and DCZ^ *p*-value							
	dcKO versus dcKO^hM3Dq and DCZ^ *p*-value							
d. Data summary of Figure 6*f*								
	Days postinjury (DPI)	−200	−150	−100	−50	0	50	100
	Kruskal–Wallis *p*-value	0.228	0.3204	0.2014	0.133	0.0066	0.005	0.0279
	LacZ^DCZ^ versus dcKO *p*-value					0.0252	0.0428	0.0428
	LacZ^DCZ^ versus dcKO^hM3Dq and DCZ^ *p*-value					0.0069	0.0069	0.0168
	dcKO versus dcKO^hM3Dq and DCZ^ *p*-value					0.0518	0.0292	0.0518

Table summarizing the statistical analysis of cFos positive neuron percentage, axon collateral index, and bouton index. Normal distribution and unpaired *t* test (a). Non-normal distribution and Kruskal–Wallis test followed by Wilcoxon rank sum exact test (b). Non-normal distribution and Kruskal–Wallis test followed by Wilcoxon rank sum exact test (c). Non-normal distribution and Kruskal–Wallis test followed by Wilcoxon rank sum exact test (d).

We then examined axon collaterals in the spinal gray matter (Fig. 6*b*). In dcKO^hM3Dq and DCZ^ mice (*n* = 7), intensities of CST collaterals in the gray matter did not differ significantly from those in LacZ^DCZ^ (*n* = 4) and dcKO (*n* = 4) mice (Fig. 6*c*, Table 5b). However, when the gray matter was separated into four regions (ipsidorsal, contradorsal, ipsiventral, and contraventral areas; Fig. 6*d*, Table 5c), the contradorsal area at 200 µm rostral to the lesion site showed significantly greater axon collaterals in the two-pronged treatment cohort (dcKO^hM3Dq and DCZ^) compared with the singular treatment group involving only genetic deletions (dcKO).

Lastly, we evaluated the number of presynaptic boutons in the spinal gray matter. The bouton indices (the amount of presynaptic structure of CSNs) in dcKO (*n* = 4) and dcKO^hM3Dq and DCZ^ (*n* = 7) mice were significantly higher than controls (LacZ^DCZ^, *n* = 4) at the lesion and caudal to the lesion site (Fig. 6*e,f*, Table 5d). At 50 µm caudal to the lesion, dcKO^hM3Dq and DCZ^ mice showed significantly higher values than dcKO mice, indicating that neural stimulation through DREADDs promotes the formation of presynaptic boutons between CSNs and spinal interneurons in the spinal gray matter after SCI.

## Discussion

Rehabilitation following SCI involves both neural regeneration and circuit formation in the affected body regions. A previous study showed that *RhoA*;*Pten* co-deletion promotes sprouting of CSNs after SCI but was insufficient to regain motor function (Nakamura et al., 2021). Other studies have shown that axon sprouting promoted by neuronal stimulation can lead to functional recovery in mice (Carmel and Martin, 2014; Brommer et al., 2021; Squair et al., 2021; Van Steenbergen et al., 2023). We hypothesized that a combinatorial approach involving both neuronal stimulation and *RhoA*;*Pten* deletion enhances axon growth and leads to motor recovery after SCI. In this study, we combined genetic deletion of *RhoA* and *Pten* in CSNs with chemogenetic stimulation of CSNs via excitatory DREADDs and examined the levels of axon regrowth and forelimb motor function after cervical SCI. Our results revealed that the synergistic effects of genetic deletion of *RhoA*;*Pten* and CSN stimulation limited axon dieback and promoted presynaptic growth in CSN axons at and below the site of injury. Furthermore, this combinatorial treatment accelerated the recovery of skilled locomotor behaviors after SCI.

An earlier study showed that mice lacking *RhoA* and *Pten* exhibited less axon dieback than controls (Nakamura et al., 2021). Hindlimb motor function was not restored in those mice (Nakamura et al., 2021). Similarly, in our current study focused on cervical SCIs, we found that genetic deletion of *RhoA* and *Pten* alone did not induce motor recovery in mice. However, when excitatory DREADDs that stimulate CSNs were combined with *RhoA*;*Pten* deletion, axon dieback was reduced to a greater extent than that in mice that had only undergone *RhoA;Pten* deletion. It is worth noting that our quantification of axon dieback relied on fluorescence intensity as a surrogate measure for axon number, which may be influenced by variations in viral injection efficiency or experimental conditions. Although the excitatory DREADDs and *RhoA;Pten* deletion promoted regrowth of injured CS axons, motor function was not fully restored. Bridging the lesion and keeping neurons in an immature state with neural stem cell (NSC) grafts (Kadoya et al., 2016; Poplawski et al., 2020; Zheng and Tuszynski, 2023), or neutralizing extracellular inhibitors at the lesion site (Houle et al., 2006; García-Alías et al., 2009; Zheng and Tuszynski, 2023), may promote greater CS axonal growth in the spinal cord. These additional treatments may be required as part of a multipronged strategy to enhance functional recovery.

Following dorsal hemisection injuries at thoracic levels in in which the CST is transected, mice lacking *RhoA* and *Pten* exhibit less axon dieback, but are still impaired in hindlimb motor control (Nakamura et al., 2021). Given the critical role that the CST plays in voluntary skilled behaviors of the forelimb, we examined forelimb motor behaviors in dcKO and dcKO with DREADD (dcKO^hM3Dq and water^ and dcKO^hM3Dq and DCZ^) mice in a grid-walking test (Schaar et al., 2010; Chao et al., 2012). The dcKO^hM3Dq and DCZ^ mice showed faster motor function recovery at 21 DPI compared with dcKO and dcKO^hM3Dq and water^ mice. Although this combinatorial approach promoted early motor recovery after SCI, full motor function was never achieved. Full restoration of motor behaviors may have been hindered for several reasons. First, the limited axon regrowth observed following SCI may be insufficient for regaining preinjury levels of motor control. Other strategies, such as NSC grafts (Kadoya et al., 2016; Poplawski et al., 2020; Zheng and Tuszynski, 2023), rehabilitation (van den Brand et al., 2012; Wang et al., 2023), or chondroitinase treatment with peripheral nerve grafts (Alilain et al., 2011; Lee et al., 2013), might further enhance motor recovery if added to the treatment regimen. A second possible explanation is that other circuits in addition to the CST, such as other descending and ascending sensory fibers, may also require restoration to regain full functionality. Finally, continuous administration of hM3Dq may result in its downregulation, which would lower the activation of CSNs (Roth, 2016). Though complete motor recovery was not observed, this study shows at least partial restoration of skilled movements following SCI.

To determine how this partial motor recovery was achieved, we examined presynaptic bouton formation in dcKO and dcKO mice with DREADDs (dcKO^hM3Dq and DCZ^). The previous study showed that *RhoA;Pten* deletion in CSNs promotes axon sprouting after SCI at thoracic levels and that synapse formation may be increased in these mice (Nakamura et al., 2021). In the present study, however, axon sprouting was absent in dcKO mice after cervical SCI, suggesting that circuit rewiring mechanisms may differ in the cervical and thoracic spinal regions. Interestingly, the addition of excitatory DREADDs that stimulate CSNs in dcKO mice caused an increase in presynaptic boutons in CST axons proximal and caudal to the cervical spinal cord lesion. Several studies have shown that excitatory DREADDs can promote presynaptic connections, while inhibitory DREADDs decrease the total number of boutons following SCI (Bradley et al., 2019; Gao et al., 2021). Therefore, presynaptic formation in CSNs may be induced by the combination of *RhoA* and *Pten* deletion coupled with neuronal activation after SCI, and this may synergistically promote motor recovery. Future motor circuit analyses using pseudorabies virus assays or electromyography after the cortical stimulation will be necessary to characterize the different stages of circuit repair promoted by this two-pronged approach.

Taken together, our results suggest that the effects of *RhoA*;*Pten* deletion combined with neuronal activation of CSNs may be a useful therapeutic approach to promote axonal growth and motor recovery after SCI. The gains in motor function observed with this two-pronged method open the door to other multifaceted treatment regimens that may elicit greater recovery of movement and functioning after SCI. Future treatment paradigms could involve a mix-and-match model tailored to the specific type and location of a SCI to deliver maximum therapeutic benefits.

## Data Availability

The datasets used and analyzed during the current study are available from the corresponding author upon reasonable request.

## Synthesis

Reviewing Editor: Jennifer Dulin, Texas A&M University Decisions are customarily a result of the Reviewing Editor and the peer reviewers coming together and discussing their recommendations until a consensus is reached. When revisions are invited, a fact-based synthesis statement explaining their decision and outlining what is needed to prepare a revision will be listed below. The following reviewer(s) agreed to reveal their identity: NONE.

Hello authors, apologies for the delay as the original reviewer did not respond to invitations to re-review. Since I have expertise in this subject area I have carefully considered the author's responses to the reviewer and I have the following comments:

1. I still have concerns about the methods for quantification of axonal dieback. In my mind, using fluorescence intensity as a surrogate measure for number of axons is not appropriate because variations in the injection efficiency or the experimental conditions can (and often do) lead to variations in the amount of neurons infected with AAV. My strong suggestion is to normalize your fluorescence intensity data to the number of fluorescently-labeled axons at the pyramidal decussation in the medulla, using transverse sections of medulla if this tissue is available. If the tissue is not available, this needs to be addressed as a caveat in the Discussion section.

2. Since the experimental subjects are mice, please change "hand" to "forepaw" throughout the manuscript.

Overall, the revised manuscript is much improved, and the addition of Figure 2 is especially useful.

## Author Response

January 14th, 2025 Jennifer Dulin, PhD Reviewing Editor eNeuro Dear Dr. Dulin, Thank you very much for sending us the reviewer's comments on our manuscript, "Modulation of extrinsic and intrinsic signaling together with neuronal activation enhance forelimb motor recovery after cervical spinal cord injury". We are grateful to the reviewer for the helpful critiques and suggestions for improving the manuscript. As noted below, we generated new data and revised the manuscript to address all the points of concern noted by the reviewer.

Major/Overall Concerns 1) Controls. The controls in this study need to be described much better. Figures suggest 3 control groups. The question arises why not compare KO+DREADDS with and w/o DCZ. Without the ligand is a good control because it takes account of any changes in the transduced cells but without activation.

Thank you so much for the suggestion. In response to this comment, we performed the axon dieback and behavioral experiments for the control and added them to the results as dcKOhM3Dq and water group. We found out that in this cohort, axon dieback was suppressed after SCI and the index had significant difference compared to LacZDCZ and dcKOhM3Dq and DCZ mice (Fig. 4). Moreover, the grid walking results showed that the dcKOhM3Dq and water group exhibited no differences in slip rates compared to the LacZDCZ group, but showed significant difference compared to dcKOhM3Dq and DCZ mice (Fig. 5).

2) Dieback analysis design. The authors have used separate controls for KO only and KO+DREADDs. Analysis (Fig 4), to my eye, shows that dieback for KO controls is less than for the KO+DREADD experiment controls. The KO control plot hits the 50% point between 100 and 200 µm, whereas the DREADD control plot does so between 200 and 300 µm. Since the measurements are based on fluorescence, requiring normalization for image intensity, the outcome will be biased by multiple factors that may have little to do with axon dieback. Also, the analysis is based on a single sagittal section; that is too few for an image intensity analysis. Using single axon counts for measurements, obviate the need for background correction. I have a related specific concern below.

We thank the reviewer for raising these important issues. In our opinion, at least three factors contribute to potential bias in the results: AAV infection efficiency, individual animal differences, and cryostat slice accuracy. We injected the same volumes of AAVs in the same locations in each animal, and used at least three animals per group. Moreover, controlling slice widths by using multiple sagittal sections will further reduce bias. Therefore, we used 3-5 sagittal images to analyze axon dieback in our experiments.

Although, in the axon dieback index plot, the 2 controls (LacZ and LacZDCZ) hit the 50% between 100 and 200 µm and between 200 and 300 µm, respectively (Fig. 4a, b), there was no significant difference between them.

We agree that using single axon counts could be an effective method for background correction in transverse sections of the spinal cord. However, in the sagittal sections, axons largely overlapped each other, and it was difficult for us to distinguish single axons, leading to inaccuracies in the analysis. Therefore, we decided not to analyze axon dieback with single axon counts in our study.

3) Collateral axon analysis design. Transverse sections were used for collateral assessment; the dieback analysis used sagittal. What is the method for collecting two planes in the same tissue. The transvers and sagittal sections are likely from different animals. This concern is related to the overall inadequacy of a general description of the overall experiments. Importantly, the method description reads: "Since the C5- specific mCherry label does not stably show mCherry distal from the lesion and made it difficult to normalize individual differences in the control mice using mCherry intensity in the grey matter, the intensity in each section was divided by the intensity in the dorsal funiculus, 1000 μm rostral from the lesion." From this description, I cannot assess the validity of the approach. Most important, the statement "does not show stability" implies use of a inadequate measure. A key outcome rests on this method.

We apologize for the confusion regarding the descriptions of our methods in the manuscript. The sagittal sections and transverse sections of the spinal cord were not from the same tissue. As shown in the new method figure (Fig. 2), the sagittal section tissues were from the long-term experiments analyzing axon dieback and grid walking, while the transverse section tissues were from the short-term experiments analyzing axon collaterals (Fig. 2c, d).

We also apologize for the confusing description of the normalization of axon collateral analyses. The mCherry intensities in the dorsal funiculus at 1000 µm rostral to the lesion were used for normalization (as was done for the axon dieback experiments) since axon dieback is limited at 1000 µm rostral to the lesion and reflects the full transduction of CST neurons.

4) Description of experimental design and animal groups. Whereas the Results section starts with design overview, this is merely a restatement of parts of the Introduction. The overall experimental design is not presented to help the reader with the Results section. This is important because design is very complex. Different controls were used for parts of the experiment. The controls should be described clearly. The authors use an impressive array of viral-based tools; the different approaches need to be clearly described in terms of why they were used and to help the reader evaluate their appropriate use. Relatedly, the groups of animals need to be described better. Figures 2 and 6 are not clear; the methods graphics are not intuitive and the timeline highlights only grid walk as an outcome (#2). I also suggest that the two table figures be combined in a way to better describe the overall project. As shown these two figures are partially/mostly redundant.

Thank you so much for raising these issues and for your helpful suggestions. We agree that the descriptions of AAV tools were insufficient in the previous version of the describes how the AAV combinations induce modifications in specific CS neurons. We also combined the two table figures and showed the timelines for the two experiments that were conducted. This is shown in our revised Figure 2.

5) How do the mechanisms of stimulation-promoted outgrowth and synapse formation intersect with RhoA and PTEN gene deletions? This is not described. Much is known about the mechanisms by which different forms of neural activation are thought to promote CST outgrowth. This can help to strengthen the underlying premise for using this particular combination.

We thank the reviewer for pointing out this issue. In our previous study (Nakamura et al., 2021), we showed that both RhoA and Pten deletion enhanced synapse formation by trans-synaptic viruses. Therefore, stimulation-promoted axon growth together with RhoA;Pten deletion may further enhance synapse formation. In our revised manuscript, we discussed this possibility.

Specific concerns Abstract -The neural stimulation method should be indicated in the abstract.

We added a statement about the chemogenetic method in the abstract.

-There is no mention of axon growth, which is an outcome.

We added a statement about axon regrowth in the abstract.

-Wording about boutons implies measurement/occurrence in cortex, but the measurements were made on corticospinal tract axons in the spinal gram matter We apologize for the insufficient descriptions in the abstract. We revised the statement to avoid this misunderstanding.

Introduction -Both chemogenetic and electrical activation are described, but nowhere is it stated in the introduction that DREADD is the stim approach being used.

We added a statement about the stimulation method in the end of our revised introduction.

-Axon growth is indicated in the closing paragraph yet it was not effectively achieved (single bin, farthest from lesion).

We revised the statement and stated that the extent was limited.

-Also, a published study used DREADDs (Yang and Martin, 2022) to promote CST axon growth.

We thank the reviewer for sharing the paper for our manuscript. We included this reference in the introduction and added it to our references section in the revised manuscript.

Methods -AAV-sources are unclear; no supplier is indicated, implying all were shared by investigators. This needs to be specified.

The supplier (Addgene) was indicated in the beginning of the AAV section in the previous version. However, as the reviewer mentioned, it was not easy to find the supplier information. We thus added the supplier's name and product numbers to each virus after the titer in our revised manuscript.

-Spinal AAV injection sites should be shown because transduction efficacy depends on injection efficacy.

We added a supplementary figure to show the injection sites in the brain and the spinal cord (Fig. 2-1).

-Cortical GFP labeled areas are shown, but we do not know how this relates to affected CSNs, which is determined by the spinal injections. This could also be shown.

We have also added this information to Supplementary Figure 2-1.

-SCI is a dorsal column cut at C5. Histology should be shown to document complete dorsal CST section, and the extent to which the cut spared projections laterally and ventrally indicated.

We added a supplementary figure to show the extent of the dorsal column lesion (Fig.

2-2). MBP antibodies were used to evaluate the completeness of the lesion. PKCγ antibodies (which stain active CS neurons) was also used to evaluate whether the CSTs were completely transected by the lesions.

-Only cFos immunohistochemistry is described; not GFAP, which is shown in Figure 4. DAPI staining is not described in the Methods. Why is GFAP so different in the examples.

Thank you so much for pointing out these issues. We have added a description of our GFAP immunohistochemistry to the results section, and DAPI staining was also added to the methods section. We believe that the reason why GFAP staining intensities seem different in each sample is due to the variations in the dorsal column lesions. Since the lesion was made manually, it was difficult to control the severity of each lesion. This may have led to the observed differences in GFAP staining.

-Quantification method of activated CSN does not make sense to me. It reads like fluorescence levels are measured in an ROI but there is no description of how double labeled neurons were detected. It is indicated that the percent of double labeled (cFos and mCherry) was determined. That would need to be done with actual counting. There is no mention of counting.

We apologize for the confusion regarding cFos quantification. We revised the wording in the methods. mCherry+ CSNs were first detected from the confocal red channel image and counted. Then the intensity of cFos within the mCherry+ CSNs was detected in the green channel and counted. Finally, the number of cFos within the mCherry+ CSNs was percentage of cFos+ neurons.

-When was DCZ injections made in relation to behavioral testing. If before testing, then the behavior outcome is not valid because it reflects a DREADD excitation effect. I do not think testing without DCZ only at the end of the experiment (Fig 5b) is a good control because there are many changes by this point, including dieback effects, bouton changes, possible CST growth, and motor experience.

Thank you for pointing out the issue regarding the DCZ injections. We added information about the injections in the revised methods. DCZ was administered twice daily after (not before) the weekly grid walking tests (Nagai et al., 2020).

-Quantification of dieback is inadequate and not convincing. First, it is not an axon count, which may be the only method with microscopic sensitivity, but fluorescence level instead. Apropos to my comments above, I am concerned that DC brightness at some unaffected rostral level has anything to do with dieback measures at the lesion site. What is the ROI? It is indicated that the measurements were taken at intervals, but the size if the sample is not indicated.

We apologize for the insufficient explanations of our axon dieback analyses. We followed the established quantification methods with some modifications (Nakamura et al., 2021). This prior study showed that the axon index decreases as it gets closer to the lesion, which reflects our results. To specify the region of interest (ROI) in the axon dieback analysis, we provided additional information in the revised methods and added a supplementary figure (Fig. 4-1). In our revised methods, we stated that the size of each ROI was set to a width of 100 µm and a height of 500 µm, and the mCherry intensity was measured within each region.

-Axon collateral measurements were made on transverse sections. This is different from dieback, which were sagittal. This must mean that separate animals were used for the dieback and collateral analysis; this was not made clear. How was the distance from lesion center ensured? Here too the measurement is mCherry fluorescence. What is C5-specific mCherry and what has this to do with the measurements? Is this referring to the AAV injection? Why is the labeling 'not stably shown? We thank the reviewer for pointing out these issues. The axon dieback and axon collateral experiments were conducted separately, and we clarified this in the revised methods and figure (Fig. 2c, d). To determine the center of the lesion, consecutive sections that likely included the lesion were first identified and the middlemost section was set as the center. This statement is added in our revised manuscript.

Regarding the question about the "C5-specific mCherry" and "not stably shown", we excluded these statements from the method and changed the statement as explained in the previous paragraph. "C5-specific mCherry" was previously used to describe axons labeled with the intersectional approach by injecting AAV8-fDIO-mCherry to the sensorimotor cortex followed by injecting AAVretro-Flpo to the spinal cord at C5. Depending on the AAV infection efficiency, the extent to which mCherry fluorescence spreads through the axon collaterals will differ in each sample. Therefore, we took the intensity in the dorsal funiculus to normalize the axon collateral amount.

Results -Key methods should be described in the Methods section; many are described in the Results.

We deleted the key methods from the results and described them in the "Experimental design" section of the revised methods.

-What is the justification for 2 daily doses. What is the expected duration of effects? The expected duration of DCZ activity was 4 hours according to a previous study (Nagai et al., 2020). We injected DCZ twice daily to prolong the stimulatory effects as long as possible.

-What is the purpose of the 'Statistical table?" I was expecting detailed results summarized there.

Thank you for pointing out the issue with the table. We have modified the tables to display the specific data and have presented them in separate files. Additionally, we have included the table legends after the reference list, as required by the guidelines.

Discussion -Some statements had citations missing. Please review to make sure all points are adequately cited.

Thank you so much for finding these errors in our manuscript. We looked through all the citations and ensured there are no further omissions.

Thank you very much for your help in processing our manuscript. We look forward to hearing your opinion.

Sincerely, The Corresponding Author and The First Author
