# Supplementary figures and images for: Modulation of Extrinsic and Intrinsic Signaling Together with Neuronal Activation Enhances Forelimb Motor Recovery after Cervical Spinal Cord Injury
Source: eNeuro. 2025 Feb 28;12(3):ENEURO.0359-24.2025. doi: 10.1523/ENEURO.0359-24.2025 (PMC11881905; doi:10.1523/ENEURO.0359-24.2025)

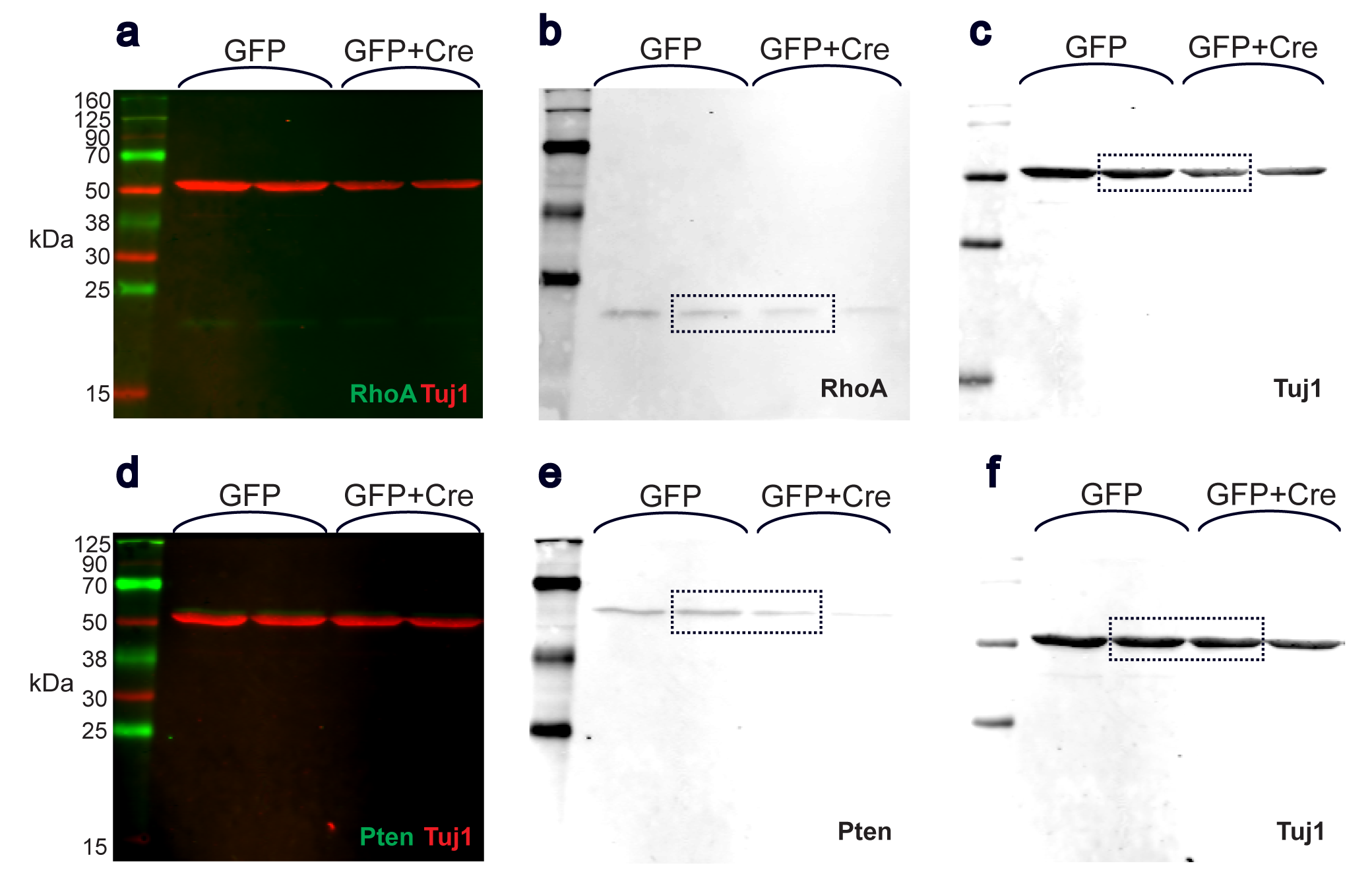

Supplement: Figure 1-1 — Genetic deletion of RhoA and Pten in the sensorimotor cortex. (a) Raw western blot image of RhoA (21 kDa) and Tuj1 (55 kDa) in the cerebral cortex of AAV-injected RhoAf/f;Ptenf/f mice. (b) Green channel image isolated from the raw image (a). (c) Red channel image isolated from the raw image (a). (d) Raw western blot image of Pten (54 kDa) and Tuj1 (55 kDa) in the cerebral cortex of AAV-injected RhoAf/f;Ptenf/f mice. (e) Green channel image isolated from the raw image (d). (f) Red channel image isolated from the raw image (d). Dotted boxes are the indicated bands for Figure 1. Download Figure 1-1, TIF file. [file eneuro-12-ENEURO.0359-24.2025-s002.tif]

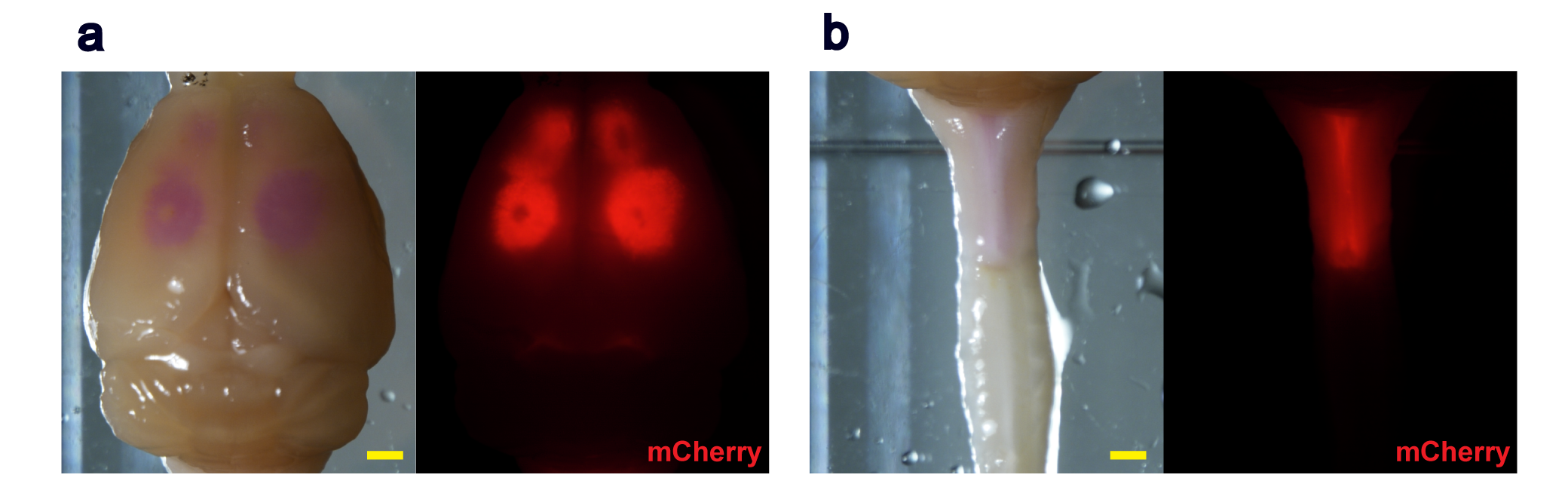

Supplement: Figure 2-1 — Stereo microscope image of brain and spinal cord after perfusion. (a-b) Raw stereo microscope image of the brain (a) and spinal cord (b) after perfusion in a dcKOhM3Dq and DCZ mouse. C5 spinal cord-specific CS neurons are visualized by mCherry fluorescence (red channel image on right). The mCherry signal is disrupted by the C5 dorsal column lesion (b). Scale bar, 1 mm. Download Figure 2-1, TIF file. [file eneuro-12-ENEURO.0359-24.2025-s003.tif]

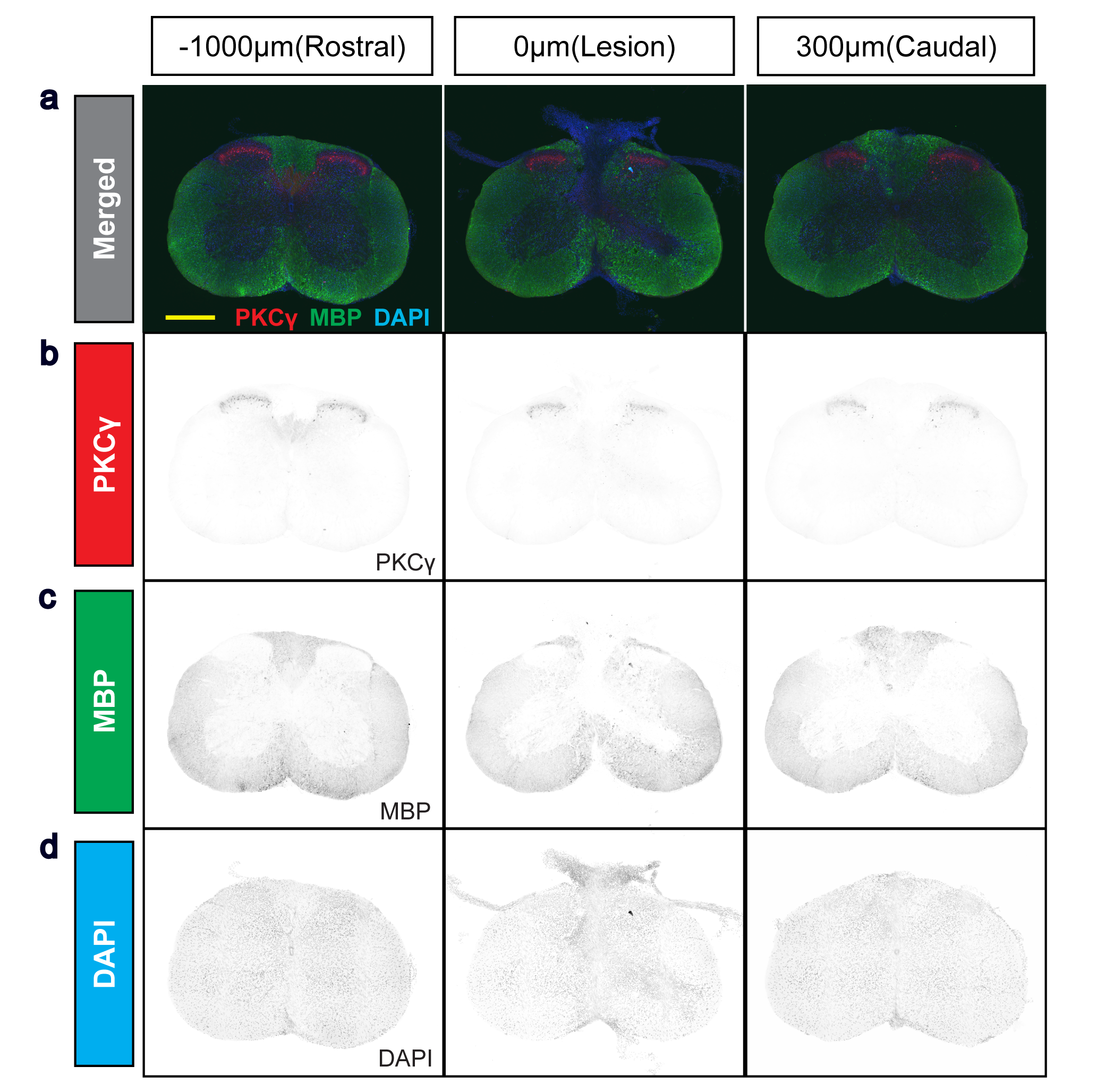

Supplement: Figure 2-2 — Confocal images of complete dorsal column lesion. (a) Merged images of PKCγ (red), MBP (green), and DAPI (blue) of spinal cords at the lesion site (0 µm), and -1000 µm (rostral) and 300 µm (caudal) to the lesion. (b-d) Individual images of PKCγ (b), MBP (c), and DAPI (d) from the merged image (a). PKCγ in the dorsal column shows the active corticospinal neurons, which fade at the lesion and caudal to the lesion (b). MBP reveals the completeness of the dorsal column lesion with minimal damage to other nervous tissue (c). Scale bar, 500 µm. Download Figure 2-2, TIF file. [file eneuro-12-ENEURO.0359-24.2025-s004.tif]

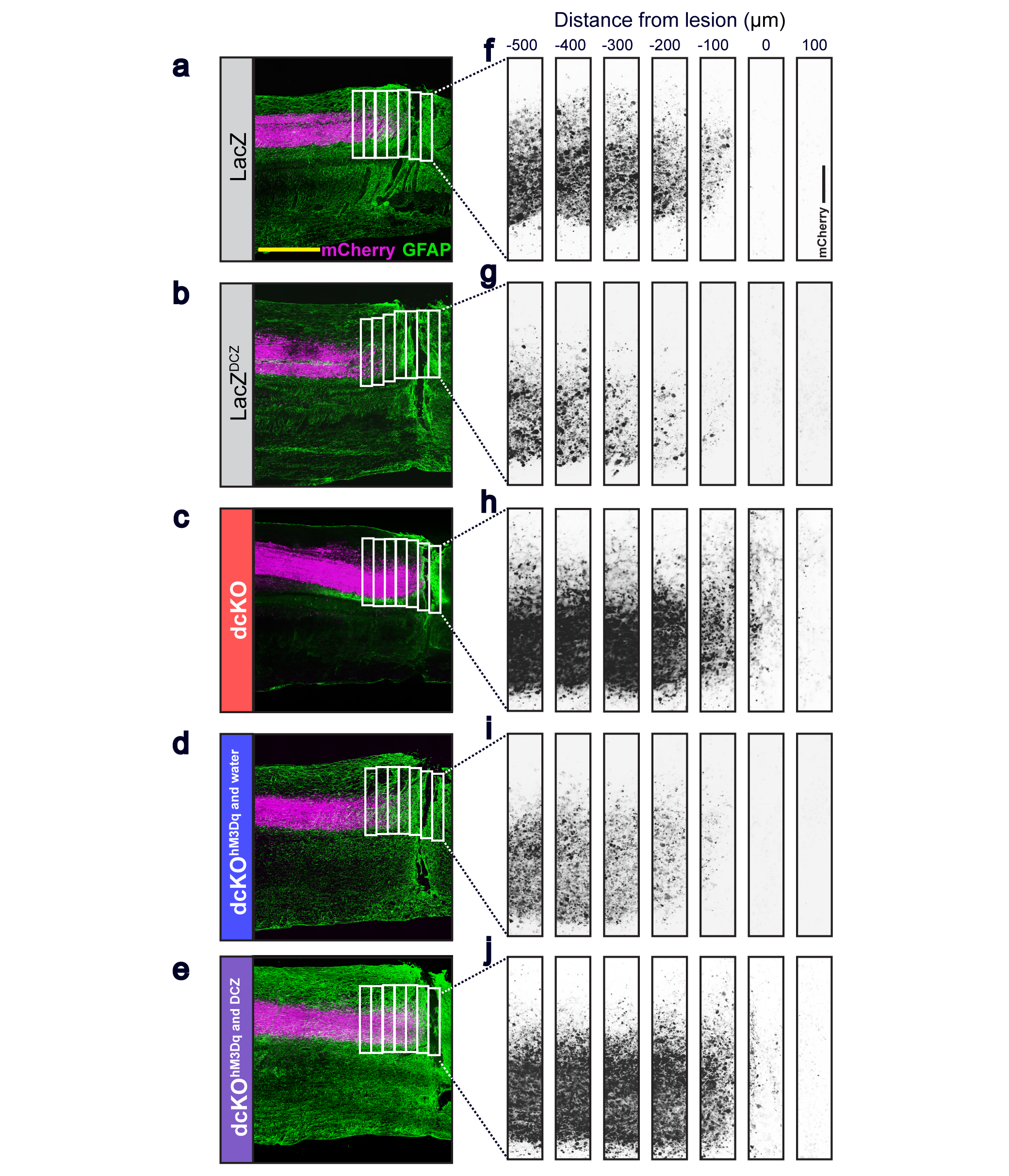

Supplement: Figure 4-1 — Region of interest in the axon dieback analysis. (a-e) Representative images of mCherry+ CST axons (magenta on the left, black on the right) and GFAP+ tissues (green on the left) in the cervical spinal cord in LacZ (a), LacZDCZ (b), dcKO (c), dcKOhM3Dq and water (d), and dcKOhM3Dq and DCZ (e) mice at 42 DPI (a, c) and 49 DPI (b, d, e). White boxes in immunofluorescence images on the left represent regions of interest for the axon dieback analysis. Scale bar, 500 µm. (f-j) Maximized mCherry+ CST axon images of the regions of interest in a-e. Scale bar, 100 µm. Download Figure 4-1, TIF file. [file eneuro-12-ENEURO.0359-24.2025-s005.tif]
